# Early sarcopterygian morphological disparity through the Devonian-Carboniferous crisis

**DOI:** 10.1038/s41598-025-31132-9

**Published:** 2025-12-10

**Authors:** Olivia Vanhaesebroucke, Olivier Larouche, Richard Cloutier

**Affiliations:** 1 Département de Biologie, Chimie et Géographie, Université du Québec à Rimouski, Rimouski, QC Canada; 2 Biology Department, Western Carolina University, Cullowhee, NC USA; 3 Center of Excellence on the Evolution of Life, Basin Studies and Applied Paleontology, Paleontological Research and Education Center, Mahasarakham University, Maha Sarakham, 44150 Thailand

**Keywords:** End-Devonian crisis, Palaeoenvironments, Palaeozoic, Geometric morphometrics, Reefs emergence, Tetrapod-like fishes, Ecology, Ecology, Evolution, Zoology

## Abstract

Major morphological adaptations characterized the early evolutionary history of sarcopterygians during the Devonian (419–359 Ma) and the Carboniferous (359–299 Ma), punctuated by environmental changes and biodiversity crises. Here we present geometric morphometrics analyses of early sarcopterygian morphology to explore the impact of environmental and phylogenetic constraints on their morphology. Three analyses were performed by digitizing 2D landmarks and semi-landmarks on palaeontological reconstructions of the body shape, cheek, and skull roof. Major changes in shape seem associated with geological and biological changes from the Late Devonian and Early Carboniferous (Mississippian). An increase in shape diversity occurred during the Middle Devonian, when the first forests appeared, influencing freshwater and transitional environments, and when reef ecosystems were the most diversified. Skull roof and cheek disparity decrease during the end-Devonian mass extinction and recover during the Mid-Late Mississippian, with the reemergence of metazoan reefs and the rise of surviving groups such as actinistians. The skull roof disparity is associated primarily with shared ancestry and appears to be largely driven by the lack of an intracranial joint in dipnoans and tetrapods. The Devonian to Carboniferous environmental changes constitute a major turning point in early sarcopterygian history by having deeply impacted their morphological disparity.

**Supplementary Information:**

The online version contains supplementary material available at 10.1038/s41598-025-31132-9.

## Introduction

Sarcopterygians are a group of diverse jawed vertebrates, with its current piscine representatives being the “lobed-finned fishes,” the actinistians (or coelacanths, two species) and lungfishes (six species), but which also includes all tetrapods, representing approximately 35,500 species^1^. The first occurrence of sarcopterygians in the fossil record dates back to the middle Silurian (Ludlow stage; 427.4–423.0 Ma), with *Guiyu oneiros* as the earliest known articulated specimen^2^. Sarcopterygians diversified during the late Silurian (427.4–419.2 Ma) and Lower Devonian (419.2–393.3 Ma), dominating this era’s vertebrate diversity along with “placoderms” (armoured fishes)^3^. During the Middle–Late Devonian (393.3–358.9 Ma), terrestrial habitats increased in complexity with the emergence of the first forests and the diversification of terrestrial arthropods, including insects^4,5^. Around that time, a new lineage of tetrapod-like fishes arose within sarcopterygians, the “elpistostegalians,” including *Panderichthys*^6^, *Qikiqtania*^7^ and *Tiktaalik*^8^. From the end of the Devonian through the Carboniferous, tetrapods greatly diversified as they colonized a spectrum of terrestrial habitats^9,10^, while piscine sarcopterygian diversity decreased^3^. This shift in sarcopterygian diversity and ecology chronologically correlates with geographic changes associated with the Devonian–Carboniferous transition, especially the Kellwasser (Frasnian–Famennian boundary, 371 Ma) and Hangenberg (Devonian–Carboniferous boundary, 359 Ma) events that constitute the end-Devonian mass extinction^11–13^. Indeed, modifications to the atmospheric composition^14^, the amplitude of the tides^15^ and sea level variations^16^, the formation of inland seas resulting from tectonic activity^11^, and the formation of large carbonate platforms^17^ all impacted the diversity of shallow aquatic habitats and may have played an important role in the diversification of early jawed vertebrates, including sarcopterygians, and their morphology^18,19^. The following Carboniferous period was a time of deep recovery, especially for the marine habitat, with the reemergence of metazoan coral reefs^20^ that created new ecological niches for the surviving sarcopterygian lineages.

The present study aims to explore the major eco-morphological modifications in the postcranial and cranial anatomy of early sarcopterygians from the Devonian and Carboniferous, and the relationships of these modifications to palaeoenvironmental data and phylogenetic relationships. As tetrapod-like sarcopterygians initiated a water-to-land transition, we expect to detect a major change in the morphospace occupation for these taxa related to their drastic change of environment^21^. Although among sarcopterygians, only tetrapods conquered the land, their aquatic close relatives are likely to exhibit a set of shared adaptations that would eventually facilitate this transition. This period is also marked by major biodiversity crises ranging from the late Middle Devonian to the Early Carboniferous. We then expect to find the general pattern of morphological disparity variation to coincide with these crisis events. Finally, early sarcopterygians inhabited various aquatic habitats, with some taxa associated with freshwater environments while others were predominantly found in marine systems^22–24^. Transitions between marine and freshwater habitats have happened several times during the evolutionary history of fishes^25,26^. Considering the chemo-physical and structural differences between these two environments^25,26^, we also expect to detect differences in morphological disparity related to the types of aquatic habitats occupied by early sarcopterygians.

## Methods

### Taxonomic diversity

**Fig. 1:**
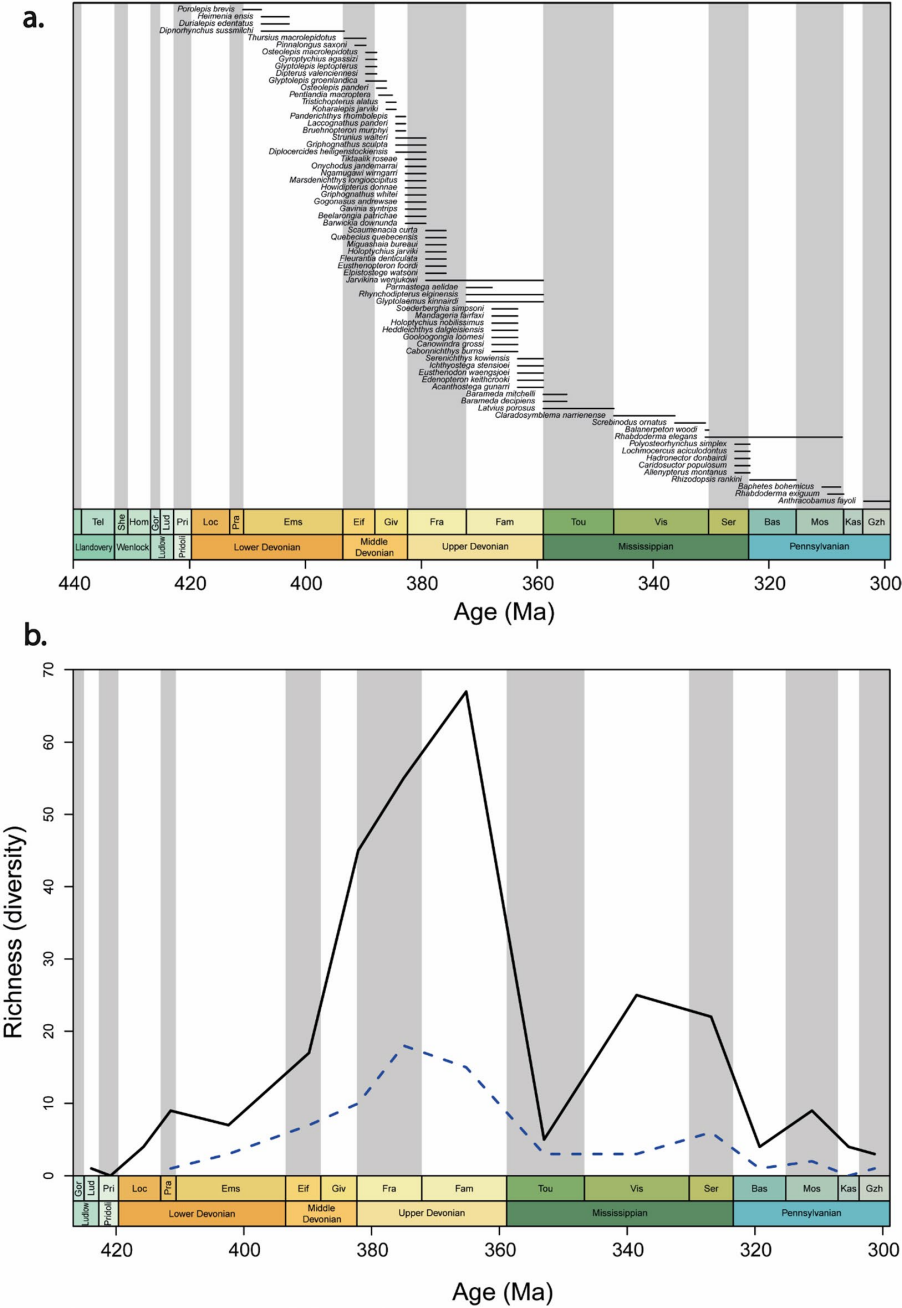
Occurrence and diversity through time of early sarcopterygian species. (**a**) Occurrence of each of the 70 species of early piscine sarcopterygians analysed; (**b**) Diversity through time of early sarcopterygians based on the richness calculated for each stage for the 70 species analyzed (dashed blue line), and 279 species of early sarcopterygian (black line). Bas: Bashkirian; Eif: Eifelian; Ems: Emsian; Fam: Famennian; Fra: Frasnian; Giv: Givetian; Gor: Gorstian; Gzh: Gzhelian; Hom: Homerian; Kas: Kasimovian; Loc: Lochkovian; Lud: Ludfordian; Mos: Moscovian; Pra: Pragian; Pri: Pridoli; Ser: Serpukhovian; She: Sheinwoodian; Tel: Telychian; Tou: Tournaisian; Vis: Viséan.

In order to get an estimate of the taxonomic diversity of early sarcopterygians during the Devonian and the Carboniferous periods, we gathered a list of 279 early sarcopterygian species from previously published databases^3,27^ and additional sources from the primary literature. For each species, the first and last occurrence dates were noted (Fig. 1, see Supplementary material Table S1 online). Our time scale sampling goes from the Ludfordian (late Ludlow, 425.6 Ma) to the late Gzhelian (Pennsylvanian, 298.9 Ma), which represents a 126.7 MY time span, and is divided into 7 epochs (Ludlow, Pridoli, Lower Devonian, Middle Devonian, Upper Devonian, Mississippian, and Pennsylvanian). We analysed sarcopterygian diversity with the R package ‘divDyn’^28^.

### Morphological dataset

Two-dimensional datasets are used rather than 3D information because the shape of fossil species is rarely conserved in three dimensions. We therefore assembled three separate two-dimensional datasets, including a total of 70 species, using palaeontological reconstructions of (1) the entire body shape (*n* = 36), (2) the cheek region (*n* = 39), and (3) the skull roof (*n* = 47) of Devonian and Carboniferous sarcopterygian species (see Supplementary Table S2 online). Only the species for which adult specimens were identified were compiled. We excluded from the analyses all species known solely from juvenile specimens (with the exception of *Serenichthys kowiensis*). Our full dataset comprises nine different groups of early sarcopterygians: Onychodontida (*n* = 2), Actinistia (coelacanths) (*n* = 12), Porolepiformes (*n* = 9), Dipnoi (*n* = 12), Rhizodontida (*n* = 4), “Osteolepiformes” (*n* = 22), “Elpistostegalia” (*n* = 2), and Tetrapoda (*n* = 7). Palaeoenvironmental data for each studied species were gathered from the literature (see Supplementary material Table S2 online) and included two variables. The first one broadly characterizes the type of aquatic habitats inhabited by early sarcopterygians and comprises three different levels along the salinity gradient: marine (*n* = 16), estuarine (*n* = 9), and freshwater species (*n* = 45). The second variable was meant to capture more precise aspects of the palaeoenvironment of each species whenever sufficient information was available. It includes 10 precise palaeoenvironments: reef (*n* = 5), coastal marine (*n* = 1), bay (*n* = 5), estuary (*n* = 10), lagoon (*n* = 8), fluvial delta (*n* = 1), alluvial plain (*n* = 7), oxbow lake and meandering river (*n* = 7), calm freshwater lake (*n* = 12), and dynamic freshwater lake (*n* = 5). Species for which no precise details on the palaeoenvironment were available were removed from this second analysis.

### Landmarking schemes

**Fig. 2:**
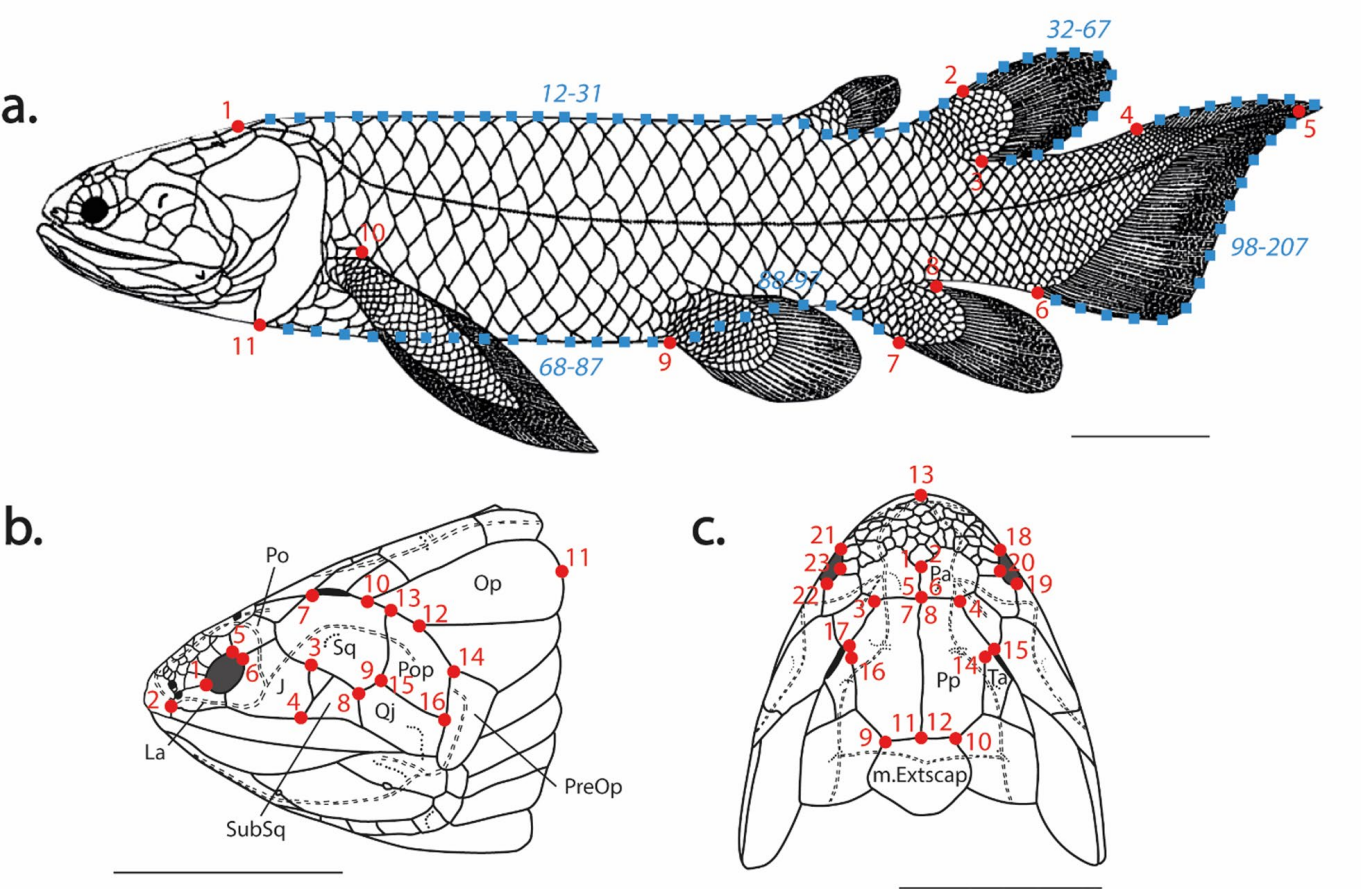
Landmarks (red circles) and semi-landmarks (blue squares) positioning scheme for geometric morphometrics analyses of (**a**) the body shape, (**b**) the cheek, and (**c**) the skull roof. *Holoptychius jarviki* (Cloutier and Schultze, 1996). See Supplementary material Table S3 for a full description of the landmarks and semi-landmarks. Scale bars, 5 cm. J: jugal; La: lacrimal; m.Extscap: median extrascapular; Op: Operculum; Pa: parietal; Po: postorbital; Pop: preopercular; Pp: postparietal; PreOp: preoperculo-submandibular; Qj: quadratojugal; Sq: squamosal; SubSq: Subsquamosal; Ta: Tabular.

We used 2D geometric morphometrics to quantify sarcopterygian morphological disparity, digitizing landmarks and semi-landmarks on palaeontological reconstructions. This approach allowed us to estimate disparity from continuous data and to obtain geometric information related to shape changes of several morphological features of early sarcopterygians. First, for the full body shape, we digitized 11 landmarks and 196 semi-landmarks describing the lateral body outline and the positioning and shapes of fins (Fig. 2a, see Supplementary material Table S3 online). Second, for the cranial shape disparity, we digitized 16 landmarks on lateral reconstructions to describe the orbital and cheek regions (Fig. 2b, see Supplementary material Table S3 online). And third, we used 23 landmarks to analyse shape variation on the skull roof (Fig. 2c, see Supplementary material Table S3 online). By capturing morphological variation in the skull from two different perspectives (dorsal and lateral), this allows us to interpret some of the major changes that occurred within this structure from a three-dimensional perspective. All landmarks and semi-landmarks were digitized using *tpsDig2* (v2.32)^29^.

Each set of landmark coordinates was rotated, translated, and scaled with a Procrustes superimposition using the *gpagen* function in ‘geomorph’ (v.4.0.1)^30^. Semi-landmarks were slid using the Procrustes distance minimisation criterion^31^. The multidimensional shape data were summarized using principal component analyses, and the first two axes of each PCA were used to build morphospaces.

### Morphological disparity analyses

We estimated morphological disparity using two different metrics suitable for multidimensional data, each reflecting different aspects of the distribution of the points in morphospaces: (1) the Procrustes variance that measures the dispersal of taxa within morphospace by providing an estimate of the difference among taxa based on the variance in the shape data, and (2) the area of convex hulls that measures the amount of global morphospace occupation for each group^32^. The latter is more sensitive to extreme values and cannot be estimated for groups with two species or less. However, it provides an intuitive metric to compare the extent of morphospace occupied by each group.

Procrustes variance was calculated for each epoch, each group of sarcopterygians, and each palaeoenvironment using the function *morphol.disparity* in the package ‘geomorph’^30^, which quantifies groupwise morphological disparity, and assesses statistical significance for each pairwise comparison^33^. Using the same set of grouping variables, the area of convex hulls in the multidimensional morphospace was estimated using a modified version of the *chull* function from the ‘grDevices’ package^34^ based on the first two axes of the PCA. We also estimated a weighted surface area based on the first five principal components of the PCA. This was done by calculating the surface area for each pair of PCs and weighting each by the combined proportion of variance explained by the two axes (see Supplementary material Fig. S1, Tables S5 and S6 online). Given that the results were qualitatively similar, we focus on the findings based on the first two PCs in the main text, as these contain most of the biologically interpretable shape changes.

As mentioned above, our time scale is divided into 7 epochs. To be independent of the time scale and to control for potential biases in this subdivision (stages are usually defined after major changes in biodiversity or environment), we also subdivided the total time interval into 7 equal time bins of 18.1 million years each. We then quantified the sum of variances for each equal time bin with the *dispRity* function in the package ‘dispRity’^35^. The resulting two patterns of morphological disparity were highly congruent. To streamline the presentation of our results, we present the morphological disparity along the epochs from the international chronostratigraphic chart in the main text. Species that overlapped two or more different epochs were classified according to their epoch of appearance.

All disparity analyses were performed in R v.4.3.2 using the packages ‘geomorph’^30^, ‘grDevices’^34^, and ‘dispRity’^35^.

### Lagerstätte effect

Lagerstätten are deposits exhibiting exceptional preservation conditions, characterized by a concentration or a preservation quality of fossils much superior to what is normally the case^36^. Several Lagerstätten are well known for the period of interest of our data sampling, such as the Gogo Formation^37^ (Late Devonian, Gogo Station, Western Australia), Escuminac Formation^22^ (Late Devonian, Miguasha, Quebec, Canada), and the Heath Formation^38^ (Mid-Carboniferous, Bear Gulch, Montana, USA). The impact of Lagerstätten on diversity and disparity estimates is referred as the “Lagerstätte effect”^39^. As most of the Mississippian species that we sampled come from the Heath Formation of Bear Gulch (Montana, USA), we investigated the potential impact of the Lagerstätte effect in our estimates by analyzing the data both with and without the Bear Gulch species.

## Results

### Body shape disparity

**Fig. 3:**
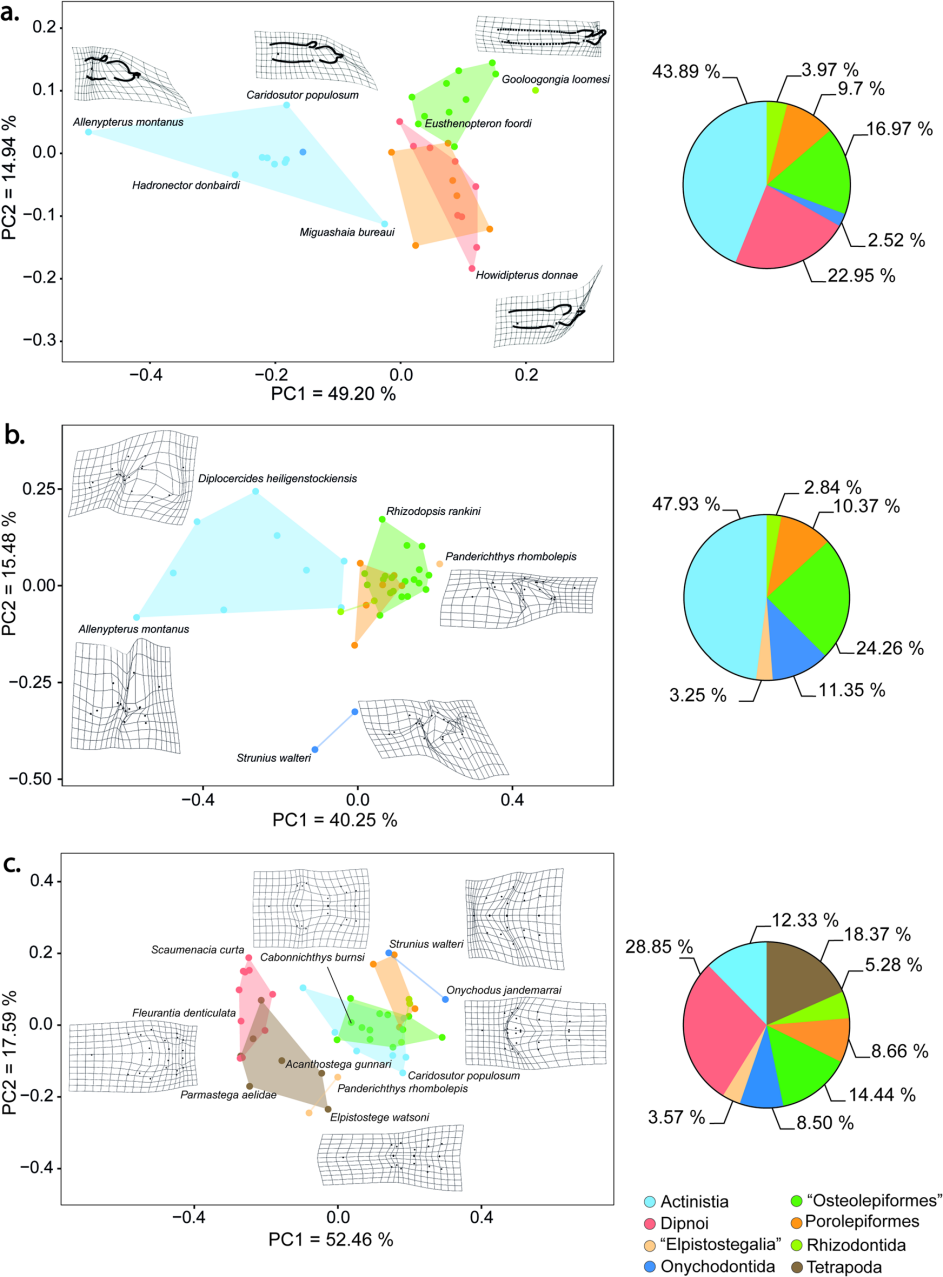
Morphological disparity of basal sarcopterygians per taxonomic group. Morphospace of principal components 1 and 2. (**a**) Body shape (*n* = 36 species); (**b**) Cheek (*n* = 39 species); and (**c**) Skull roof (*n* = 47 species). Deformation grids show the differences between the extreme forms along each axis and the mean shape. The pie charts illustrate the proportion of disparity each group represents based on their respective Procrustes variance. If categories include only one species, it will be represented by a single dot.

The first axis of the PCA (PC1) explains 49.20% of the variation among 36 species, and contrasts species with shortened bodies and a tri-lobed caudal fin, such as the actinistian *Allenypterus montanus*, to species with more fusiform body shapes with a heterocercal or diphycercal caudal fin, such as in the rhizodont *Gooloogongia loomesi* (Fig. 3a). PC1 also separates the Actinistia from the remaining sarcopterygians (Fig. 3a). Actinistians occupy a relatively larger area in the morphospace than the other groups, having the largest convex hull surface area (see Supplementary material Table S4 online), and representing 43.89% of the total disparity.

**Fig. 4:**
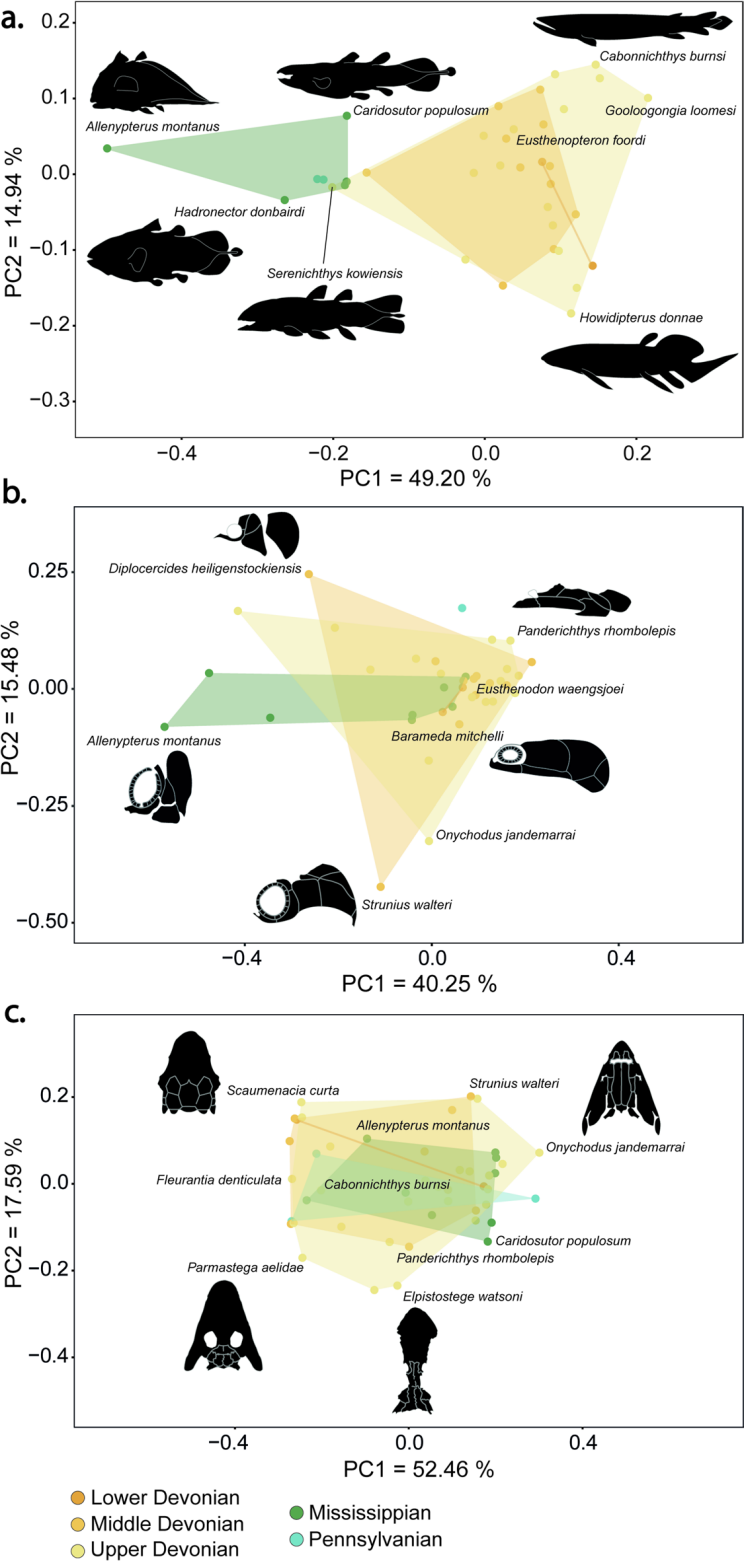
Morphological disparity of basal sarcopterygians per geological epoch. Principal component analysis showing PC1 versus PC2 disparity based on 2D geometric morphometrics. (**a**) Body shape (*n* = 36 species); (**b**) Cheek (*n* = 39 species); and (**c**) Skull roof (*n* = 47 species). If categories include only one species, it will be represented by a single dot.

PC1 also clearly discriminates Devonian from Carboniferous species (Fig. 4a). Frasnian sarcopterygians represent 17.55% of the total disparity, the Famennian species 20.44%, while the Serpukhovian piscine sarcopterygians represent 35.53%. The Upper Devonian and Mississippian constitute the epochs with most of the body shape disparity among early sarcopterygians (see Supplementary material Table S7 online). Body shape disparity increased during the beginning of the Middle Devonian (Eifelian) reaching a maximum during the Middle Devonian (Givetian) and the Upper Devonian (Famennian). Body shape disparity then decreases towards the end of the Carboniferous (Fig. 6).

**Fig. 5:**
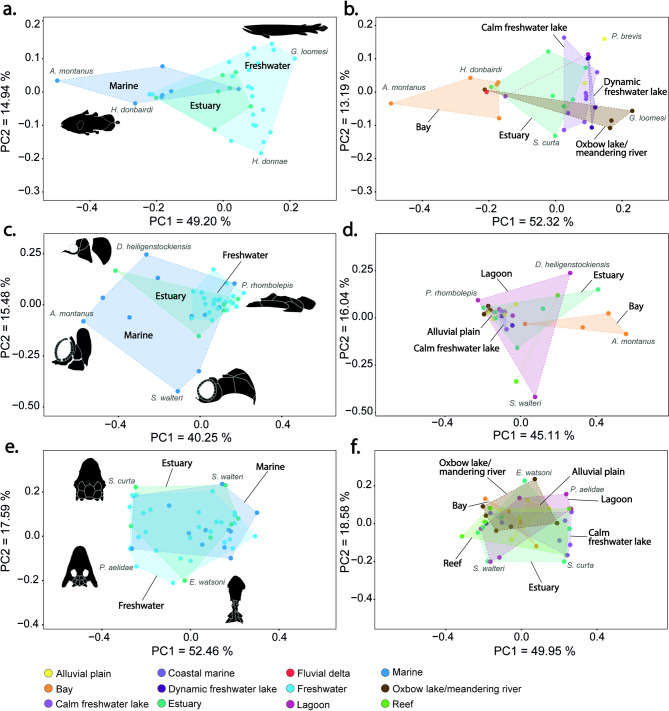
Morphological disparity of basal sarcopterygians per aquatic habitats. Morphospace of principal components 1 and 2. (**a**–**b**). Body shape; (**c**–**d**). Cheek; (**e**–**f**). Skull roof. **b**, **d**, and **f** represent more precise palaeoenvironments and exclude species with no detailed information about it. If categories include only one species, it will be represented by a single dot.

**Fig. 6:**
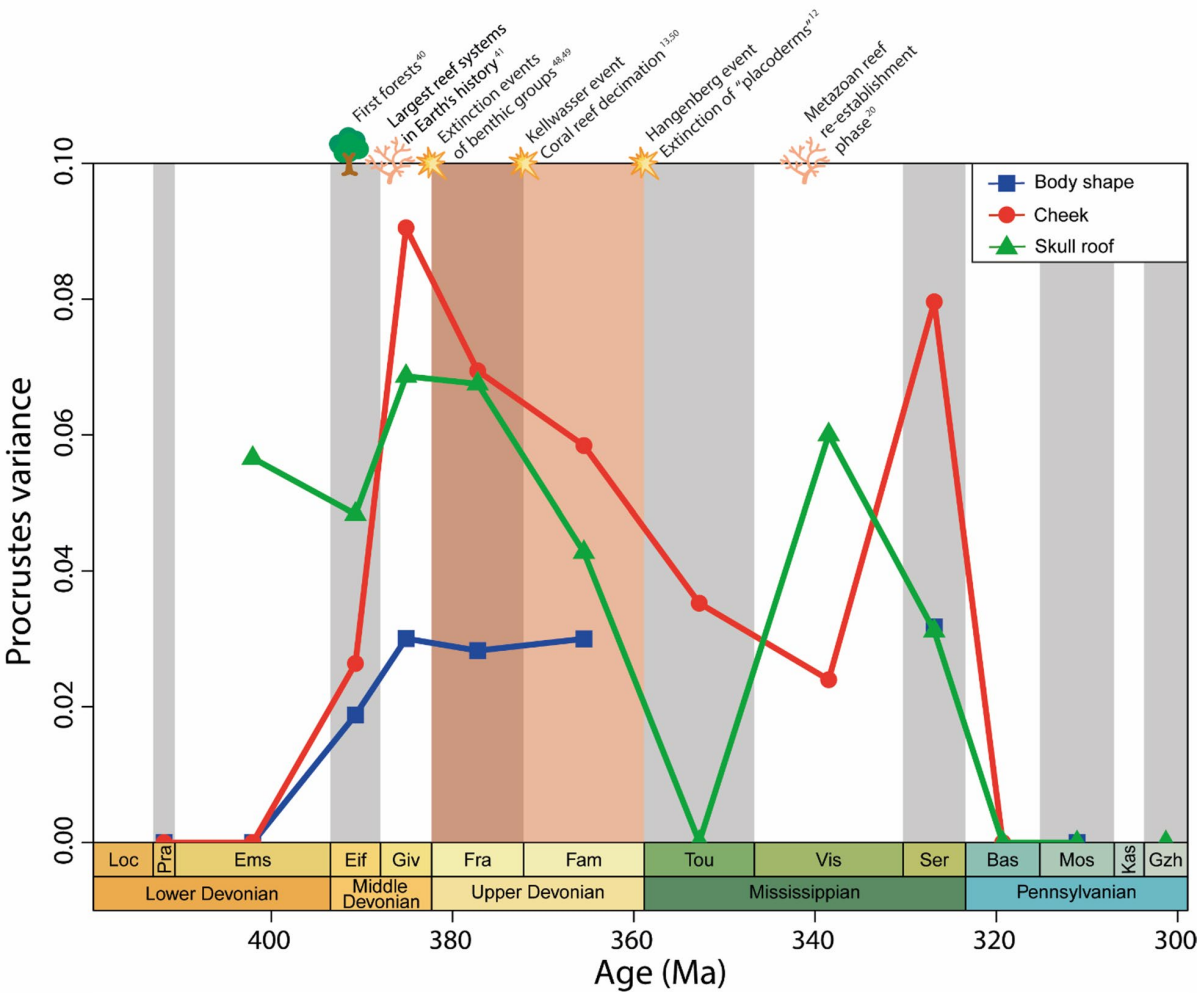
Procrustes variance for body shape (blue squares), cheek (red dots), and skull roof (green triangles) disparity through time. Major Devonian and Carboniferous geological and biological events are represented.

Finally, PC1 presents a gradient from the marine species mostly concentrated on the left half of the morphospace to the freshwater species that mostly occupy the right half, with estuarine species overlapping both spaces (Fig. 5a). Freshwater species represent nearly half of the total disparity (49.04%) (see Supplementary material Table S7 online). Looking at the palaeoenvironment of early sarcopterygians on a finer scale, PC1 seems to separate the bay environment representing 33.54% of the total disparity from all other palaeoenvironments (Fig. 5b, see Supplementary material Table S7 online).

PC2 explains 14.94% of the variation and represents mostly a difference in the shape of the tail. The tail may be heterocercal, such as in the dipnoan *Howidipterus donnae*, whereas *Cabonnichthys burnsi* and other “Osteolepiformes” are characterized by a diphycercal tail (Fig. 3a).

### Cheek disparity

PC1 explains 40.25% of the morphological variation of the cheek region among sarcopterygians. This represents a shift from a deeper and shorter skull, such as in the actinistian *Allenypterus montanus*, to a more flattened and elongated skull as in the “elpistostegalian” *Panderichthys rhombolepis* (Fig. 3b). PC2 explains 15.48% of the skull variation, representing differences in the position and size of the opercular series. At one extremity of the axis, the opercular series is narrow and dorsally positioned as in the onychodont *Strunius walteri*, whereas at the other extremity the opercular series is wider as in the actinistian *Diplocercides heiligenstockiensis*. The distinction between the skulls of Devonian and Carboniferous species is less pronounced than for full body shape, especially along PC1 (Fig. 4b). Devonian species are spread across the entire morphospace exhibiting substantial cheek disparity. Carboniferous species are mostly differentiated among themselves by the level of elongation of their skulls. However, the change in the shape of sarcopterygian skulls seems to be better explained by shared ancestry; the different groups of sarcopterygians being separated in the morphospace (Fig. 3b). Again, actinistians stand out from the rest of sarcopterygians, being characterized by shorter and deeper heads. They exhibit higher levels of cheek disparity with a Procrustes variance significantly higher than all the other groups (0.0961, *p* < 0.05), and by occupying a larger area of the morphospace (see Supplementary material Tables S4 and S7 online). They account for 47.93% of the total cheek disparity across sarcopterygians. This pattern is similar to that of the full body shape disparity. The Onychodontida are also clearly separated from the rest with narrower and more dorsally positioned opercular series, a characteristic that most likely reflects the plesiomorphic condition of the cheek region in basal actinopterygians. Finally, the Dipnomorpha and Tetrapodomorpha are closely packed and are characterized by more elongated and flattened heads.

Looking at the aquatic palaeoenvironments and habitats (Fig. 5c–d, see Supplementary material Tables S4 and S7 online), marine species occupied a larger area in the morphospace. Marine species accounted for more than half of the total cheek disparity (52.73%) and had a significantly higher Procrustes variance (0.1297, *p* < 0.001) than the freshwater species (0.0397, *p* < 0.001). Freshwater species are densely clustered, exhibiting a more flattened and elongated cheek region. Lagoonal, estuarine, and bay species seem more spread out across the morphospace (Fig. 5d, see Supplementary material Tables S4 and S7 online).

### Skull roof disparity

Among the three morphological systems studied, the skull roof displays the greatest disparity. Indeed, PC1 explains 52.46% of the variation in skull roof shape among early sarcopterygians (Fig. 3c), representing primarily differences in the shape and position of parietals and postparietals. They vary from elongated and anteriorly positioned as in the onychodont *Onychodus jandermarrai*, to shortened and posteriorly positioned as in the dipnoan *Fleurantia denticulata.* PC2 explains 17.59% of the variation. It represents a change in the width of the skull roof accompanying the migration of the eyes from a lateral position as in the onychodont *Strunius walteri*, to a more dorsal and median position in the tetrapod *Elpistostege watsoni*.

Even if Late Devonian species seem to present a higher morphological disparity (Fig. 4c, see Supplementary material Table S7 online), there is no obvious change between the skull roof shapes of Devonian and Carboniferous species (Fig. 4c). Similarly, no major change in shape is detected among the palaeoenvironments (Fig. 5e–f). However, when focusing on the disparity among sarcopterygian groups, obvious differences can be observed among three of them (Fig. 3c). The first group consists of the Actinistia, Onychodontida, Porolepiformes, Rhizodontida, and “Osteolepiformes” that form a tight cluster towards the right portion of the morphospace. Here as well, the Actinistia appears to be a highly disparate group, exhibiting the broadest distribution in shape space (Fig. 3c, see Supplementary material Table S4 online). The second group is the “Elpistostegalia” characterized by a shift of the orbits towards the top of the skull; they occupy the lower portion of the morphospace and overlap with the Tetrapoda. Finally, the third grouping is the Dipnoi and Tetrapoda clustered in the left portion of the morphospace, which are characterized by a shortening of the posterior (otico-occipital) region of the skull roof.

### Early sarcopterygian diversity and morphological disparity variation through time

The diversity of sarcopterygians presents two successive peaks of maximum richness, the first during the Famennian and the second during the Mid-Late Mississippian (Viséan) (Fig. 1b). A decrease in sarcopterygian richness is observed between the Devonian and the Carboniferous. In all three analyses, morphological disparity for each age was estimated as the sum of Procrustes variances (Fig. 6). Body shape disparity increases during the Middle Devonian before it stabilises during the Upper Devonian. Skull roof and cheek disparity seem to follow a similar pattern. A first maximum is reached during late Middle Devonian and early Upper Devonian, which corresponds to the time of appearance of the first forests on land, and the peak of coral reef ecosystems^40,41^. A second maximum is reached during the Middle and Upper Mississippian, which appears to correspond to the time of recovery of metazoan reefs^20^ (Fig. 6). Both skull roof and cheek disparities decrease simultaneously with the Upper Devonian biodiversity crises. A small discrepancy is observed between the first morphological disparity maximum during the Givetian and the maximum species diversity during the Famennian (Figs. 1b and 6).

### Lagerstätte effect

The morphological disparity results excluding the Bear Gulch species are presented in the supplementary information (see Supplementary material Fig. S2 online). The major trends of disparity remain the same, although patterns of cheek disparity seem to be impacted by the absence of these Mississippian species, which include three actinistians from our database. Indeed, when excluding the species from Bear Gulch, the cheek disparity peak of the Serpukhovian is no longer present, and may therefore represent a consequence of a Lagerstätte effect, which suggests that the disparity peak observed during the Serpukhovian in our all-species analyses could be influenced by the presence of the Bear Gulch Lagerstätte species and could be the result of a Lagerstätte effect.

## Discussion

The main goal of this study was to explore the patterns of morphological disparity (body shape, cheek region, and skull roof) among Devonian and Carboniferous sarcopterygians, and to explore changes in overall disparity through time. The main source of body shape disparity opposed elongated and fusiform morphologies to shorter and deeper body shapes. The cheek region showed a shift from deeper and shorter skulls to flattened and elongated ones, as well as some variation in the position of the opercular series. Finally, the major sources of disparity for the skull roof were variation in width, as well as in the position of the eyes, parietal, and postparietal bones. These observed patterns were found to be explained in part by shared ancestry within the major groups of sarcopterygians, the geological epochs, and habitat types.

### Devonian environmental changes influenced the early diversification of sarcopterygians

The Devonian and Carboniferous periods are punctuated by major climatic, geological and biological events that could have impacted sarcopterygian history. The first maximum of both sarcopterygian morphological disparity and species richness dates to the Middle–Upper Devonian. During the Middle Devonian, reefs proliferated and occupied the largest marine surface area in Earth’s history^41,42^. Concurrently on land, the first forests appeared^40^ and progressively transformed the terrestrial landscape, which was accompanied by the diversification of several groups of terrestrial invertebrates^4,5,43^. The increased presence of plants on land also led to a change in the atmospheric composition, decreasing CO_2_ levels and increasing O_2_ levels^14^, both of which are associated with global cooling^14^. Simultaneously, these changes facilitated an increase in the organic matter input from continental waters, especially along the continental shelves^44^. Moreover, the fluctuation in the Laurussia ice cap covering induced by the variation of global temperature generated changes in the sea level and the formation of epicontinental shallow seas^16,45^. These events have been associated with the timing of early diversification of new sarcopterygian lineages such as the first tetrapod-like fishes, likely during the early-middle Devonian^9,46^. During the Devonian, sarcopterygians dominated the jawed vertebrate diversity along with “placoderms”^3^ and the use of different niches by new sarcopterygian lineages could explain the increase in morphological disparity that we observed.

This period is also characterized by an increase of tectonic activity associated with volcanic activity^45^, which led to the opening of the Paleotethys ocean and the formation of large carbonate platforms at collision sites^45^. These major changes deeply impacted the aquatic living forms and led to several biodiversity crises during the Upper Devonian that contributed to the Late Devonian mass extinction^47^. From a biological point of view, structural benthic groups such as rugose and tabulate corals^48^ and stromatoporoids^49^ were lost during the first crisis at the end of the Givetian (Middle Devonian). The second remarkable crisis, known as the Kellwasser event (Frasnian–Famennian boundary, 371 Ma), is characterized by an abundance of black shales originating from a high level of organic matter deposition (originating from the continental environment), underlying large scale anoxic conditions^13^. This consequential anoxia event in aquatic habitats could have led to the coral reef decimation by the end of the Frasnian (Upper Devonian), with 80% of tabulate corals^50^ and 60% of rugose coral genera^50^ going extinct along with 80% of ostracod species^51^ and more than 75% of brachiopod species^50^, all of which played a key role in Devonian trophic networks^52^. Finally, the Hangenberg event marking the Devonian–Carboniferous boundary^12^ (359 Ma), is characterized by the loss of several fish groups^3^. Particularly, Sallan and Coates^3^ described a crash in the diversity of piscine sarcopterygians and “placoderms” at the Devonian–Carboniferous boundary^3^, constituting a major turn-over in the aquatic organisation because of their dominant place among vertebrates during the Devonian, when they occupied a wide range of ecological niches^53,54^. The loss of several piscine sarcopterygian groups during these successive crises^3^ accompanying the loss of several ecological niches could help explain the observed decrease of both skull roof and cheek disparity during the Upper Devonian.

### The aquatic habitats influenced cheek disparity

The sarcopterygian cheek region showed a shift from deeper and shorter skulls to flattened and elongated ones, as well as some variation in the position of the opercular series. In fishes, the cheek region corresponds to the region extending laterally from the orbit to the posterior extremity of the skull, including the opercular series. The cheek region plays structural and functional roles in the jaw suspension and is an important muscle attachment site for several jaw muscles, but is also involved in the structural support and flexibility of the head^55^ and associated kinematics of breathing and feeding. This region is therefore deeply involved in the diversity of jaw movements and ecological adaptation of fishes^55^. This could explain the strong response of the cheek disparity facing the fall of several aquatic systems with the end-Devonian crisis.

Focusing on the influence of the type of aquatic habitats, we observed low cheek morphological disparity in freshwater species despite their taxonomic richness. Indeed, when considering cheek shape, marine species appeared significantly more disparate than freshwater sarcopterygians. The aquatic environment constitutes a highly complex system of various habitats and environmental constraints^56^. Differences in physical characteristics between freshwater and marine aquatic habitats constitute a potential driver for morphological diversification within fishes^25,57,58^. The differences in the patterns of morphological disparity of the cheek region between marine and freshwater fish species have never been studied before. However, more general patterns associated with other aspects of fish diversification could provide additional insights to this investigation. First of all, marine and freshwater systems share common structural and functional characteristics, which have led to similar ecologies of their respective communities despite their independent evolutionary histories^59,60^. The feeding apparatus tends to have similar adaptations in freshwater and marine systems that are dependent on organismal dietary preferences^61^. Given that the cheek bones are functionally involved with the jaws and gill rakers, differences in feeding strategies likely explain some of the morphological variation in this region among marine fishes. Moreover, in our analyses, most of the marine species were actinistians and dipnoans, groups for which the increase in the cheek region variation corresponds to the period directly following the Devonian^62^. These two groups were predominantly found in marine waters and in coastal systems (e.g. estuaries, deltas, bays) which went through important changes during the early Carboniferous. The re-emergence of the coral reefs during the Viséan (Middle Mississippian) complexified the marine system, whereas the early Carboniferous coasts were dominated by delta and estuary systems due to the end-Devonian—Early Carboniferous marine transgressions^63,64^. This could have offered new ecological opportunities to these two groups, triggering their ecomorphological diversification during their recovery period following the end-Devonian mass extinction. As for the lack of variation of the cheek region for the freshwater species, this could be indicative of a decoupling between the early diversification of the freshwater sarcopterygians and their subsequent ecological diversification in terms of trophic habits. It has also been shown that, contrary to the actinopterygian lineage, early sarcopterygians tend to lose skull bones mainly through fusion of elements^65^. This tendency could have led to a more stable and less modular skull in the derived sarcopterygian lineages close to the transition from freshwater systems to land^65,66^. The specific paleoenvironmental categories used in this study involve relatively few taxa, which may introduce sampling biases into the comparisons. The observed patterns remain meaningful, but these limitations should be considered when interpreting the results. A more robust analysis could be performed in future work, by using a broader sampling to test the stability of these trends.

### Shared ancestry influenced skull roof disparity

Unlike the cheek region, skull roof disparity did not seem to be profoundly influenced by the type of aquatic system, which could suggest little or no ecological constraints associated with these habitats. Moreover, no clear change was detected for skull roof shape between the Devonian and the Carboniferous. The main sources of disparity for the skull roof were variation in width, as well as in the position of the eyes, parietal, and postparietal bones, and was best explain by shared ancestry. An elongation and a decreasing depth of the skull was observed within the “Osteolepiformes” and Porolepiformes. However, this is without generating a substantial increase in disparity within these groups, despite their high species richness. Looking at jaws, Troyer et al.^67^ also detected low morphological disparity and low evolutionary rates among tetrapodomorphs, which could indicate few morphological changes during the initial stages of terrestrialisation. In taxa such as extant crocodilians, it has been suggested that such modifications of the skull roof shape could be advantageous for lateral snapping, particularly in aquatic habitats or transitional zones^21^. This could explain the observed changes of the skull roof in early tetrapods and their close relatives that were most likely primarily aquatic, despite displaying some adaptations facilitating the progressive use of terrestrial habitats^66^. Another striking example of this is the dorsal migration of the eyes accompanying the flattening and elongation of the preorbital region, and the shortening of the parietal and postparietal shields. In these semi-aquatic animals, dorsally oriented eyes could have allowed them to spot prey on land during a time of major diversification of terrestrial plants and invertebrate groups^4,68,69^. The last remarkable result concerning the skull roof disparity was the segregation of dipnoans and tetrapods from the other groups along the first axis of the PCA, opposing a skull with an elongated preorbital region (such as in tetrapods and dipnoans) with a skull more elongated in the post-orbital region. In all early sarcopterygians, an intracranial joint is located on the top of the skull, separating the ethmosphenoid and the otoccipital regions^70^. The intracranial joint is thought to increase the biting force and could have facilitated the processing of large-sized prey^70^. This structure is absent in dipnoans and tetrapods, which have a fused neurocranium^71^. This could explain why these two groups form a tight cluster, separated from the other lineages, in our skull roof analyses.

### Post-crisis ecological opportunities influenced body shape disparity

Contrary to what has been observed for cheek and skull roof disparity, body shape disparity seems to remain stable across the end-Devonian crises. Fish overall body shape is most closely linked to locomotory patterns^72^. The changes associated with the end-Devonian crises may have had minimal impacts on potential ecological opportunities that would trigger new types of locomotion, which could explain this stability in sarcopterygian postcranial shape through the crises. However, Sallan and Galimberti^73^ reported an overall reduction in body sizes for most of the fish lineages following the Devonian crisis. Thus, the selective pressures during this period, especially the change in the atmosphere, may have been more consequential for organismal size, and less so for body shapes. This may also suggest a more resilient response from body shape facing these crises compared to the skull, which could be more strongly impacted by ecological constraints associated with feeding and breathing^74^.

The following Carboniferous period was a time of deep recovery^75^ especially with the re-emergence of coral reef ecosystems during the Viséan that created new ecological niches in marine habitats^20^. Reefs are particularly important for the structure of marine ecosystems, providing more structurally complex habitats, and have already been shown to be tightly linked to the diversification of fishes^76–78^. Despite the apparent stability of body shape morphological disparity, a major shift in body shape of early sarcopterygians is observed between the Devonian and Early Carboniferous forms. The main source of body shape disparity among early sarcopterygians opposed more elongated and fusiform morphologies to shorter and deeper body shapes. This pattern is similar to those observed among actinopterygians^79^ and could suggest a conserved first axis of body morphological diversification among osteichthyans.

In our analyses, we found this shift to also correlate to the rise of actinistians during the Early Carboniferous, which generated new shapes among piscine sarcopterygians. Indeed, Clement et al.^27^ demonstrated a rapid increase in rates of morphological evolution in actinistians during the Carboniferous (around the Viséan) and in all our analyses, actinistians appear to be the most disparate group of early sarcopterygians, accounting for nearly half of the total morphological disparity for the complete body shape. Moreover, actinistians exhibit distinct body shapes from the other sarcopterygians. Although actinistians are often referred to as “living fossils” and are proposed as a model of bradytelic evolution^80,81^, the early history of actinistians is marked by a dramatic change in their morphology^27,67,82–84^. In their recent preprint, Rivero-Vega et al.^84^ detected successive peaks of morphological diversification in the early history of actinistians before their decline during the Mesozoic. Basal actinistians retained a shape closer to that of the other sarcopterygians (lungfishes and porolepiforms), characterised by a heterocercal caudal fin and a more elongated postorbital region^82^, while Carboniferous and Palaeozoic actinistians expanded into entirely new regions of the morphospaces. This could explain the position in morphospace of the Devonian *Miguashaia*^27,82^ near the other sarcopterygian groups in the analyses compared to the other actinistians. *Miguashaia* appears closer to a generalised sarcopterygian shape rather than a later-branching actinistian shape, characterised by a tri-lobed tail such as in the extant *Latimeria*^27,83^. Before their body shape stabilised during the Mesozoic, some early actinistians exhibited unusual shapes such as in the Carboniferous *Allenypterus montanus*^27,82,83^. The high morphological disparity of actinistians during the Palaeozoic is even more remarkable when compared to other groups such as the “Osteolepiformes” which represented the largest group in all our analyses and did not present comparable levels of morphological disparity. Troyer et al.^67^ also found actinistians to exhibit high disparity and elevated rates of jaw morphological change compared to other groups of sarcopterygians. The periods during which the diversity of actinistians increased have been correlated to abiotic factors such as tectonic activity and changes in ocean levels^27^ and also seem to correspond to the re-emergence of corals systems during the Viséan^20,41^. Thus, the biogeography and ecological opportunities following the end-Devonian crises could have contributed to the morphological diversification of actinistians, found in habitats ranging from lagoons to reefs, during the Carboniferous.

## Conclusion

The mid-Palaeozoic era was a crucial time for sarcopterygian evolutionary history, marked by tremendous ecological changes triggered by the tectonic activity and atmospheric modifications associated with the Late Devonian. We found variation patterns of sarcopterygian morphological disparity to be cohesive with the main ecological events happening throughout this era, notably the extinction and re-emergence of coral reefs, which play an important structural role in marine ecosystems and fish diversification. Actinistians appeared to be the most disparate group of early sarcopterygians, which provides additional support for the early radiation of actinistians to have been accompanied by high ecomorphological disparity. We found a similar pattern of morphological disparity variation for the skull roof and the cheek region, with a decrease during the end-Devonian mass extinction, followed by a recovery period during the Mid-Late Mississippian. We found evidence that the increase in cheek morphological disparity during the Mississippian might have been associated with a Lagerstätte effect from the Bear Gulch species, but as our dataset includes several Lagerstätten from different ages, the effect of one of them in particular might be minimal. Furthermore, we found cohesive triggering ecological factors during the Mississippian that could support this disparity. While this study provides valuable insights on the morphological diversification of early branching sarcopterygian lineages, a deeper study of the morphological disparity among early tetrapodomorphs could bring additional information regarding the morphological innovations attributed to the water-to-land transition.

## Supplementary Information

Below is the link to the electronic supplementary material.

Supplementary Material 1

## Data Availability

Data and code are available on GitHub: https://github.com/oliviavanhaesebroucke/Early-sarcopterygian-disparity.git.
