# Supplementary material for: Early sarcopterygian morphological disparity through the Devonian-Carboniferous crisis
Source: Sci Rep. 2025 Dec 10;16:1623. doi: 10.1038/s41598-025-31132-9 (PMC12800246; doi:10.1038/s41598-025-31132-9)
Supplement: Supplementary file 1 — Supplementary Material 1 [file 41598_2025_31132_MOESM1_ESM.docx]

**Scientific Reports**

**Supplementary information for**

**Early sarcopterygian morphological disparity through the Devonian-Carboniferous crisis**

Olivia Vanhaesebroucke^1*^ (olivia.vanhaesebroucke@uqar.ca), Olivier Larouche^2^ (olarouche@email.wcu.edu) and Richard Cloutier^1,3^ (richard_cloutier@uqar.ca)

^1^ Laboratoire de Paléontologie et Biologie évolutive, Département de biologie, chimie et géographie, Université du Québec à Rimouski, Rimouski, Québec, Canada, G5L 3A1

^2^Western Carolina University, College of Arts and Sciences, Biology Department, Cullowhee, North Carolina, United States of America

^3^ Center of Excellence on the Evolution of Life, Basin Studies and Applied Paleontology; Paleontological Research and Education Center, Mahasarakham University, Maha Sarakham 44150, Thailand

*****Author for correspondence

**Table S1.** List of the 279 basal sarcopterygian species included in diversity analyses. FAD (first appearance datum) corresponds to the first time the species appears in the fossil record, and LAD (last appearance datum) to the last time it appears.

| Species | Epoch | | Age | Formation/Country | References | FAD | LAD | |
| --- | --- | --- | --- | --- | --- | --- | --- | --- |
| Silurian | | | | | | | | |
| *Guiyu oneiros* | Ludlow | | Ludfordian | Kuanti Formation, Qujing, Yunnan, China | Zhu *et al.* 2009 | 425,6 | 423 | |
| Devonian | | | | | | | | |
| *Acanthostega gunarri* | Upper Devonian | | Famennian | Britta Dal Formation, Gauss Halvø, East Greenland | Coates 1996; Clack *et al*. 2012; Jarvik 1952 | 363,33 | 358,9 | |
| *Adelargo schultzei* | Upper Devonian | | Famennian | Hunter Siltstone, Hervey Group, Grenfell, New South Wales, Australia | Sallan & Coates 2010; Johanson & Ritchie 2000; Young 1999 | 363,33 | 358,9 | |
| *Andreyevichthys epitomus* | Upper Devonian | | Famennian | Andreyevka-2, Khovanshshina Beds, Zovalzkh Formation, Tula, Siberia, Russia | Sallan & Coates 2010; Lebedev *et al*. 2018; Krupina 1987 | 363,33 | 358,9 | |
| *Aztecia mahalae* | Middle Devonian | | Givetian | Aztec Siltstone, Antarctica | Sallan & Coates 2010; Johanson & Ahlberg 2001 | 386,03 | 384,36 | |
| *Barwickia downunda* | Upper Devonian | | Frasnian | Mt. Howitt, Victoria, Australia | Long & Clement 2009; Long 1992 | 382,7 | 379,2 | |
| *Beelarongia patrichae* | Upper Devonian | | Frasnian | Mt. Howitt, Victoria, Australia | Long & Clement 2009; Long 1987 | 382,7 | 379,2 | |
| *Bruehnopteron murphyi* | Middle Devonian | | Givetian | Red Hill, Northern Simpson Park Range, Nevada, USA | Schultze & Reed 2012 | 384,36 | 382,7 | |
| *Cabonnichthys burnsi* | Upper Devonian | | Famennian | Mandagery Sandstone, Canowindra, New South Wales, Australia | Ahlberg & Johanson 1997 | 367,76 | 363,33 | |
| *Canowindra grossi* | Upper Devonian | | Famennian | Mandagery Sandstone, Canowindra, New South Wales, Australia | Long 1985; Thomson 1973 | 367,76 | 363,33 | |
| *Chagrinia enodis* | Upper Devonian | | Famennian | Chagrin shale, Ohio, USA | Schaeffer 1962; Schaeffer 1962 | 372,2 | 358,9 | |
| *Cheirodipterus onawayensis* | Middle Devonian | | Givetian | Onaway Stone Quarry, Presque Isle County; Michigan, USA | Sallan & Coates 2010; Schultze 1982 | 386,03 | 384,36 | |
| *Chirodipterus australis* | Upper Devonian | | Frasnian | Gogo Formation, Australia | Sallan & Coates 2010; Miles 1977 | 382,7 | 379,2 | |
| *Chirodipterus potteri* | Upper Devonian | | Famennian | MandagerySandstone, Canowindra, New South Wales, Australia | Sallan & Coates 2010; Kemp 2000 | 367,76 | 363,33 | |
| *Chirodipterus wildungensis* | Upper Devonian | | Frasnian | Bad Wildungen, Germany | Sallan & Coates 2010; Gross 1933 | 375,7 | 372,2 | |
| *Coelacanthus welleri* | Upper Devonian | | Famennian | Maple Mill Shale, Kinderhook Limestone Series, Burlington, Iowa, USA | Forey 1998; Eastman 1908 | 372,2 | 358,9 | |
| *Conchodus elkneri* | Middle Devonian | | Givetian | Gerolstein, Germany; Zone II, Wietrznia, Kadzielnia, Holy Cross Mountains, Poland | Sallan & Coates 2010; Gorisdro-Kulczyska 1950 | 387,7 | 363,33 | |
| *Conchodus jerofejewi* | Upper Devonian | | Frasnian | Stolbovo, Dubniki beds, Sargayevian region stage, Russia | Sallan & Coates 2010; Pander 1858 | 379,2 | 375,7 | |
| *Conchodus ostreaformis* | Upper Devonian | | Frasnian | Scaat Craig beds, Upper Old Red Sandstone, Highland, Scotland | Sallan & Coates 2010; McCoy 1848 | 375,7 | 372,2 | |
| *Conchodus parvulus* | Upper Devonian | | Famennian | Fossil Ridge, Parting Formation, Gunnison County, Colorado, USA | Sallan & Coates 2010; Bryant & Johnson 1936 | 363,33 | 358,9 | |
| *Densignathus rowei* | Upper Devonian | | Famennian | Red Hill, Catskill Formation, Pennsylvania, USA | Sallan & Coates 2010; Daeschler 2000 | 363,33 | 358,9 | |
| *Diabolepis speratus* | Lower Devonian | | Lochkovian | Qujing, Yunnan, China | Chang & Yu 1984 | 413,6 | 409,73 | |
| *Dictyonosteus arcticus* | Middle Devonian | | Givetian | West Spitzbergen, Norway | Forey 1998; Stensiö 1918 | 387,7 | 382,7 | |
| *Diplocercides heiligenstockiensis* | Middle Devonian | | Givetian | Bergisch-Gladbach, Nordrhein-Westfalen, Germany | Jessen 1966; Schultze & Cloutier 1996 | 384,36 | 379,2 | |
| *Diplocercides jaekeli* | Upper Devonian | | Frasnian | Chagrin shale, Ohio, USA | Forey 1998; Stensio 1922 | 382,7 | 372,2 | |
| *Diplocercides kayseri* | Upper Devonian | | Frasnian | Bad Wildungen, Germany; Kadzielnia Quarry and Wietrznia Quarry in Kielce, Poland | Schultze & Cloutier 1996; von Koenen 1895 | 375,7 | 363,33 | |
| *Diplocercides* sp*.* | Upper Devonian | | Frasnian | Central Iran | Janvier 1974 | 382,7 | 372,2 | |
| *Diplocercides* sp*.* | Upper Devonian | | Famennian | Kadzielnia Quarry, Kielce, Poland | Szrek 2007 | 367,76 | 363,33 | |
| *Dipnorhynchus sussmilchi* | Lower Devonian | | Emsian | Taemas-Wee Jasper/Burrinjuck limestones of New South Wales, Australia | Clement *et al*. 2016; Etheridge 1906 | 407,6 | 393,3 | |
| *Dipterus calvani* | Middle Devonian | | Givetian | Cedar Valley Limestone, Milwaukee Formation, Iowa | Sallan & Coates 2010; Eastman 1900 | 384,36 | 375,7 | |
| *Dipterus ithacensis* | Upper Devonian | | Frasnian | Rhinestreet shale, Portage Formation, New York, USA | Sallan & Coates 2010; Williams 1881 | 375,7 | 372,2 | |
| *Dipterus johnsoni* | Upper Devonian | | Famennian | Fossil Ridge, Parting Formation, Chaffee Group, Gunnison County, Colorado, USA | Sallan & Coates 2010; Bryant & Johnson 1936 | 363,33 | 358,9 | |
| *Dipterus murchisoni* | Upper Devonian | | Frasnian | Zone II, Wietrznia, Kadzielnia, Holy Cross Mountains, Poland | Sallan & Coates 2010 | 372,2 | 363,33 | |
| *Dipterus nelsoni* | Upper Devonian | | Famennian | Chemung facies/Venago Formation, Warren, Pennsylvania, USA | Szrek 2007; Newberry 1889 | 363,33 | 358,9 | |
| *Dipterus uddeni* | Middle Devonian | | Givetian | Cedar Valley Limestone, Milwaukee Formation, Iowa, USA | Sallan & Coates 2010; Eastman 1900 | 384,36 | 375,7 | |
| *Dipterus valenciennesi* | Middle Devonian | | Eifelian | Achanarras fish beds, Caithness flagstone, Orcadian Basin, Scotland | Ahlberg & Trewin 1995 ; Challands 2015 ; Dineley & Metcalf 1999 (p171); Sedgwick & Murchison 1829 | 389,56 | 387,7 | |
| *Duffichthys mirabilis* | Upper Devonian | | Frasnian | Scat Craig Beds, Elgin, Scotland | Ahlberg 1992 | 375,7 | 372,2 | |
| *Durialepis edentates* | Lower Devonian | | Emsian | Klerf Formation, Westphalia, Germany | Mondéjar-Fernández *et al*. 2021; Otto 2007 | 407,6 | 402,83 | |
| *Edenopteron keithcrooki* | Upper Devonian | | Famennian | Boyds Tower and Hegarty bay, New South Wales, Australia | Young *et al*. 2019; Young *et al*. 2013 | 363,33 | 358,9 | |
| *Elginerpeton pancheni* | Upper Devonian | | Frasnian | Scaat Craig beds, Upper Old Red Sandstone, Scotland | Sallan & Coates 2010; Ahlberg 1995 | 375,7 | 372,2 | |
| *Elpistostege watsoni* | Upper Devonian | | Frasnian | Escuminac Formation, Miguasha, Quebec, Canada | Cloutier *et al*. 2020; Westoll 1938 | 379,2 | 375,7 | |
| *Eoactinistia foreyi* | Lower Devonian | | Pragian | Fairy Formation, Buchan, Victoria, Australia | Johanson *et al*. 2006; Johanson *et al*. 2006 | 409,73 | 407,6 | |
| *Eoctenodus* sp*.* | Middle Devonian | | Givetian | Aztec Siltstone, Antarctica | Sallan & Coates 2010; Hills 1929 | 386,03 | 384,36 | |
| *Euporosteus eifeliensis* | Middle Devonian | | Givetian | Gerolstein, Germany | Gross 1950; Schultze & Cloutier 1996; Jaekel 1927 | 384,36 | 382,7 | |
| *Euporosteus yunnanensis* | Lower Devonian | | Pragian | Posongchong Formation, Zhaotong, Yunnan Province, China | Zhu *et al*. 2012; Lu *et al*. 2012 | 408,66 | 407,6 | |
| *Eusthenodon gavini* | Upper Devonian | | Famennian | Hunter Siltstone, Hervey Group, New South Wales, Australia | Sallan & Coates 2010; Johanson & Ritchie 2000 | 363,33 | 358,9 | |
| *Eusthenodon waengsjoei* | Upper Devonian | | Famennian | Britta Dal Formation on Gauss, East Greenland | Blom *et al*. 2007; Jarvik 1952 | 363,33 | 358,9 | |
| *Eusthenopteron foordi* | Upper Devonian | | Frasnian | Escuminac Formation, Miguasha, Quebec, Canada | Schultze & Cloutier 1996; Whiteaves 1881 | 379,2 | 375,7 | |
| *Eusthenopteron jenkinsi* | Upper Devonian | | Frasnian | Fram Formation, Nunavut Territory, Canada | Downs *et al*. 2018 | 382,7 | 379,2 | |
| *Eusthenopteron kurshi* | Upper Devonian | | Frasnian | Lode, Gauja Formation, Latvia | Sallan & Coates 2010; Zupins 2008 | 382,7 | 379,2 | |
| *Fleurantia denticulata* | Upper Devonian | | Frasnian | Escuminac Formation, Miguasha, Quebec, Canada | Cloutier 1996; Graham-Smith & Westoll 1937 | 379,2 | 375,7 | |
| *Ganorhynchus* sp*.* | Upper Devonian | | Frasnian | Chemung facies/Venago Formation, Warren, Pennsylvania, USA | Sallan & Coates 2010; Traquair 1873 | 363,33 | 358,9 | |
| *Gavinia syntrips* | Upper Devonian | | Frasnian | Avon River Group, Mount Howitt, Victoria, Australia | Long 1999 | 382,7 | 379,2 | |
| *Glyptolaemus kinnairdi* | Upper Devonian | | Famennian | Dura Den, Upper Old Red Sandstone, Scotland | Cloutier (pers. Com); Huxley 1861 | 372,2 | 358,9 | |
| *Glyptolepis baltica* | Middle Devonian | | Givetian | Amata Regional Stage, Luga river bassin, Leningrad Region, Russia; Gauja Regional Stage, Lode Quarry, Latvia | Gross 1936 | 384,36 | 379,2 | |
| *Glyptolepis ?dellei* | Upper Devonian | | Famennian | Plavsk Regional Stage, Russia |  | 367,76 | 363,33 | |
| *Glyptolepis groenlandica* | Middle Devonian | | Eifelian | Canning Land, Vilddal Group, East Greenland | Jarvik 1972 | 389,56 | 386,03 | |
| *Glyptolepis leptopterus* | Middle Devonian | | Eifelian | Lethen Bar and Tynet Burn, Nairn, Old Red Sandstone, Scotland | Agassiz 1844 ; Newman 2010 ; Dineley & Metcalf 1999 (p171) | 389,56 | 387,7 | |
| *Glyptolepis ?nessovi* | Middle Devonian | | Givetian | Aruküla Regional Stage, Luga river bassin, Leningrad Region, Russia |  | 387,7 | 386,03 | |
| *Glyptolepis paucidens* | Middle Devonian | | Givetian | Orcadian Basin, Scotland | Agassiz 1844 | 387,7 | 386,03 | |
| *Glyptolepis quadrata* | Middle Devonian | | Eifelian | Narova Regional Stage, Luga river bassin, Leningrad Region, Russia | von Eichwald 1844; Ivanov & Lebedev 2011 | 393,3 | 387,7 | |
| *Glyptolepis* sp*.* | Middle Devonian | | Eifelian | Narova Regional Stage, Luga river bassin, Leningrad Region, Russia | Ivanov & Lebedev 2012; Agassiz 1843 | 393,3 | 387,7 | |
| *Glyptolepis* sp*.* | Middle Devonian | | Givetian | Aruküla Regional Stage, Luga river bassin, Leningrad Region, Russia | Ivanov & Lebedev 2012; Agassiz 1843 | 387,7 | 386,03 | |
| *Glyptolepis* sp*.* | Upper Devonian | | Frasnian | Amata Regional Stage, Luga river bassin, Leningrad Region, Russia | Ivanov & Lebedev 2012; Agassiz 1843 | 382,7 | 379,2 | |
| *Glyptolepis* sp*.* | Upper Devonian | | Frasnian | Plavinas Regional Stage, Luga river bassin, Leningrad Region, Russia | Ivanov & Lebedev 2012; Agassiz 1843 | 382,7 | 379,2 | |
| *Glyptopomus bystrowi* | Upper Devonian | | Famennian | Ketleri Formation, Latvia; Turgenevo Formation of the Plavsk Regional Stage, Russia | Sallan & Coates 2010; Zupiņš 2009 | 363,33 | 358,9 | |
| *Glyptopomus elginensis* | Upper Devonian | | Famennian | Rosebrae beds, Upper Old Red Sandstone, Morayshire, Scotland | Sallan & Coates 2010; Jarvik 1950 | 372,2 | 358,9 | |
| *Gogodipterus paddyensis* | Upper Devonian | | Frasnian | Gogo Formation, Australia | Sallan & Coates 2010; Miles 1977 | 382,7 | 379,2 | |
| *Gogonasus andrewsae* | Upper Devonian | | Frasnian | Gogo Formation, Australia | Hu *et al*. 2019; Long 1985 | 382,7 | 379,2 | |
| *Gooloogongia loomesi* | Upper Devonian | | Famennian | Canowindra fish beds, New South Wales, Australia | Johanson & Ahlberg 1998 ; Cloutier *et al*. 2020 (supp.) | 367,76 | 363,33 | |
| *Griphognathus minutidens* | Middle Devonian | | Givetian | Lode Formation, Gauja, Latvia | Sallan & Coates 2010; Gross 1956 | 384,36 | 382,7 | |
| *Griphognathus sculpta* | Middle Devonian | | Givetian | Upper Plattenkalk, Heiligenstock quarry, Bergisch-Gladbach, Germany | Friedman 2007; Schultze 1969 | 384,36 | 379,2 | |
| *Griphognathus whitei* | Upper Devonian | | Frasnian | Gogo Formation, Australia | Campbell & Barwick 2002; Pridmore & Barwick 1993; Miles 1977 | 382,7 | 379,2 | |
| *Grossipterus crassus* | Middle Devonian | | Givetian | Lode Formation, Gauja, Latvia | Sallan & Coates 2010; Gross 1933 | 384,36 | 382,7 | |
| *Gyroptychius agassizi* | Middle Devonian | | Eifelian | Orcadian Basin, Scotland | Newman & Dean 2010 ; Dineley & Metcalf 1999 (p171); Traill 1841 | 389,56 | 387,7 | |
| *Gyroptychius milleri* | Middle Devonian | | Eifelian | Caithness, Orcadian Basin, Scotland; Aruküla Formation, Estonia | Sallan & Coates 2010; Jarvik 1948 | 389,56 | 386,03 | |
| *Hamodus lutkevitshi* | Middle Devonian | | Givetian | Karski outcrop, Harma substage, Estonia | Obruchev 1933 | 387,7 | 382,7 | |
| *Heddleichthys dalgleisiensis* | Upper Devonian | | Famennian | Dura Den Formation, Midland Valley, Scotland | Snitting 2009; Anderson 1859 | 367,76 | 363,33 | |
| *Heimenia ensis* | Lower Devonian | | Emsian | Verdalen Member, Wood bay Formation, Spitsbergen, Norway | Mondéjar-Fernández & Clément 2012; Ørvig 1969 | 407,6 | 402,83 | |
| *Heimenia* sp*.* | Lower Devonian | | Emsian | Podlazie, Holy Cross Mountians, Poland | Wilk 2023; Wilk *et al* 2022 | 402,83 | 393,3 | |
| *Heliodus lesleyi* | Upper Devonian | | Famennian | Chemung facies/Venago Formation, Warren, Pennsylvania, USA | Sallan & Coates 2010; Newberry 1875 | 363,33 | 358,9 | |
| *Holodipterus elderae* | Upper Devonian | | Frasnian | Gogo Formation, Australia | Sallan & Coates 2010; Pridmore 1994 | 382,7 | 379,2 | |
| *Holodipterus gogoensis* | Upper Devonian | | Frasnian | Gogo Formation, Australia | Sallan & Coates 2010; Miles 1977 | 382,7 | 379,2 | |
| *Holodipterus kiprijanowi* | Upper Devonian | | Famennian | *Cheiloceras* beds, Holy Cross Mountains, Poland; Turgenevo Formation, Plavsk Regional Stage, Russia | Sallan & Coates 2010; P&er 1858 | 372,2 | 358,9 | |
| *Holodipterus meemanae* | Upper Devonian | | Frasnian | Gogo Formation, Australia | Sallan & Coates 2010; Pridmore *et al*. 1994 | 382,7 | 379,2 | |
| *Holopterygius nudus* | Upper Devonian | | Frasnian | Heiligenstock quarry, Bergisch-Gladbach, Germany | Hartkopf-Fröder *et al*. 2004 ; Friedman & Coates 2006; Jessen 1973 | 382,7 | 379,2 | |
| *Holoptychius bergmanni* | Upper Devonian | | Frasnian | Fram Formation, Ellesmere Island, Nunavut, Canada | Downs *et al*. 2013 | 382,7 | 375,7 | |
| *Holoptychius flemingi* | Upper Devonian | | Famennian | Dura Den Formation, Midland Valley, Scotland | Agassiz 1844; Cloutier & Schultze 1996 | 367,76 | 363,33 | |
| *Holoptychius giganteus* | Upper Devonian | | Frasnian | Scaat Craig beds, Upper Old Red Sandstone, Highland, Scotland | Agassiz 1843, 1844, 1845 | 375,7 | 372,2 | |
| *Holoptychius halli* | Upper Devonian | | Famennian | Catskill Formation, Delhi, New York, USA | Newberry 1889; Eastman 1908 | 363,33 | 358,9 | |
| *Holoptychius jarviki* | Upper Devonian | | Frasnian | Escuminac Formation, Miguasha, Quebec, Canada | Cloutier & Schultze 1996 | 379,2 | 375,7 | |
| *Holoptychius nobilissimus* | Upper Devonian | | Famennian | Dura Den, Upper Old Red Sandstone, Scotland | Dineley & Metcalf 1999; Agassiz 1839 | 367,76 | 363,33 | |
| *Holoptychius* sp*.* | Upper Devonian | | Frasnian | Kap Kolthoff Group, Greenland | Jarvik 1942; Blom *et al*. 2007 | 382,7 | 379,2 | |
| *Holoptychius* sp*.* | Upper Devonian | | Frasnian | Nadmeshi beds, Latvia | Mondéjar-Fernández & Meunier 2020; Agassiz 1839 | 382,7 | 372,2 | |
| *Holoptychius* sp*.* | Upper Devonian | | Famennian | Khovanshchinian Regional Stage, Andreyevka-2 locality, Tula Region, Russia | Alekseev *et al*. 1994; Agassiz 1839 | 363,33 | 358,9 | |
| *Holoptychius* sp*.* | Upper Devonian | | Famennian | Ketleri outcrop, Ketleri Formation, Venta River, Latvia | Lukševičs & Zupins 2004 | 363,33 | 358,9 | |
| *Holoptychius* sp*.* | Middle Devonian | | Givetian | Aruküla Regional Stage, Luga river bassin, Leningrad Region, Russia | Ivanov & Lebedev 2011; Agassiz 1839 | 387,7 | 386,03 | |
| *Holoptychius* sp*.* | Upper Devonian | | Frasnian | Amata Regional Stage, Luga river bassin, Leningrad Region, Russia | Ivanov & Lebedev 2011; Agassiz 1839 | 382,7 | 379,2 | |
| *Hongyu chowi* | Upper Devonian | | Famennian | Zhongning Formation, Quarry at Shixiagou, Qingtongxia, Ningxia, China | Zhu *et al*. 2017 | 372,2 | 358,9 | |
| *Howidipterus donnae* | Upper Devonian | | Frasnian | Mt. Howitt, Victoria, Australia | Long & Clement 2009 | 382,7 | 379,2 | |
| *Howidipterusus santacrucensis* | Upper Devonian | | Famennian | Zone II, Wietrznia, Kadzielnia, Holy Cross Mountains, Poland | Sallan & Coates 2010 | 367,76 | 363,33 | |
| *Howittichthys warrenae* | Upper Devonian | | Frasnian | Mt Howitt, Victoria, Australia | Long & Holl& 2008 | 382,7 | 379,2 | |
| *Hyneria lindae* | Upper Devonian | | Famennian | Red Hill, Duncannon Member, Catskill Formation, Hyner, Pennsylvania | Sallan & Coates 2010; Thompson 1968 | 363,33 | 358,9 | |
| *Hynerpeton bassetti* | Upper Devonian | | Famennian | Red Hill, Duncannon Member, Catskill Formation, Hyner, Pennsylvania, USA | Sallan & Coates 2010; Daeschler *et al*. 1994 | 363,33 | 358,9 | |
| *Ichthyostega eigili* | Upper Devonian | | Famennian | Britta Dal Formation, Celsius Bjerg Group, East Greenland | Sallan & Coates 2010; Säve-Söderbergh 1932 | 363,33 | 358,9 | |
| *Ichthyostega stensioei* | Upper Devonian | | Famennian | East Greenland, Aina Dal Formation | Blom 2005; Säve-Söderbergh 1932 | 363,33 | 358,9 | |
| *Iowadipterus halli* | Middle Devonian | | Givetian | Cedar Valley Limestone, Milwaukee Formation, Iowa | Sallan & Coates 2010; Schultze 1992 | 384,36 | 375,7 | |
| *Iranorhynchus seyedemamii* | Upper Devonian | | Givetian | Kerman, Tabas Region, eastern Iran | Sallan & Coates 2010; Janvier & Martin 1978 | 384,36 | 379,2 | |
| *Jakubsonia livnensis* | Upper Devonian | | Famennian | Gornostayevka quarry, SW of the Livny Town, Oryol Region, Russia | Sallan & Coates 2010; Lebedev 2004 | 372,2 | 367,76 | |
| *Jarvikia arctica* | Upper Devonian | | Famennian | Aina Dal Formation and Britta Dal Formation, Celsius Bjerg Group, East Greenland | Sallan & Coates 2010; Lehman 1959 | 363,33 | 358,9 | |
| *Jarvikia lebedevi* | Upper Devonian | | Famennian | Tugenevo beds/Dankoc-Lebedyan beds, Zadonckian/Plavsk Regional stage, central Devonian field, Oryol, Metsensk district, Russia | Sallan & Coates 2010; Krupina | 372,2 | 358,9 | |
| *Jarvikina wenjukowi* | Upper Devonian | | Frasnian | Upper Variegated Series, Yukhora village, Leningrad Oblast, Russia | Cloutier *et al*. 2020 (supp.); Rohon 1889 | 379,2 | 358,9 | |
| *Jemalongia ritchiei* | Middle Devonian | | Givetian | Hervey Group, Cloghnan Shale Formation, Jemalong Weir, Forbes, New South Wales, Australia | Young 2024 | 387,7 | 372,2 | |
| *Kenichthys campbelli* | Middle Devonian | | Eifelian | Chuandong Fonnalion of Qujing, Yunnan, China | Chang & Zhu 1993 | 389,56 | 386,03 | |
| *Koharalepis jarviki* | Middle Devonian | | Givetian | Mount Crean, Lashly Range, Antarctica | Young *et al*. 1992; Cloutier *et al*. 2020 (supp.) | 386,03 | 384,36 | |
| *Laccognathus embryi* | Upper Devonian | | Frasnian | Fram Formation, Ellesmere Island, Nunavut, Canada | Downs *et al*. 2011 | 382,7 | 375,7 | |
| *Laccognathus grossi* | Middle Devonian | | Givetian | Gauja Formation, Latvia | Vorobyeva 2006 | 384,36 | 382,7 | |
| *Laccognathus panderi* | Middle Devonian | | Givetian | Lode, Gauja Formation, Latvia | Vorobyeva 2006; Gross 1941 | 384,36 | 382,7 | |
| *Langliera socqueti* | Upper Devonian | | Famennian | Evieux Formation, Famenne, Belgium | Sallan & Coates 2010; Clément *et al*. 2009 | 363,33 | 358,9 | |
| *Latvius deckerti* | Middle Devonian | | Givetian | Upper Plattenkalk, Heiligenstock quarry, Bergisch-Gladbach near Cologne, Nordrhein-Westfalen (North Rhine-Westphalia), Germany | Jessen 1966 | 384,36 | 379,2 | |
| *Latvius grewingki* | Upper Devonian | | Frasnian | Snetnaya Gora beds, Russia | Gross 1933; Trinajstic *et al*. 2019 | 382,7 | 379,2 | |
| *Latvius niger* | Middle Devonian | | Givetian | Bergish Gladbach, Oberer Plattenkalk, Germany | Sallan & Coates 2010; Jessen 1973 | 384,36 | 379,2 | |
| *Latvius obrutus* | Upper Devonian | | Famennian | Ketleri outcrop, Ketleri Formation, Venta River, Latvia | Sallan & Coates 2010; Vorobyeva 1977 | 363,33 | 358,9 | |
| *Litoptychius bryanti* | Upper Devonian | | Famennian | Fossil Ridge, Parting Formation, Chaffee Group, Gunnison County, Colorado, USA | Sallan & Coates 2010; Denison 1951 | 363,33 | 358,9 | |
| *Livoniana multidentata* | Middle Devonian | | Givetian | Lode Formation, Gauja, Latvia | Sallan & Coates 2010; Ahlberg *et al*. 2000 | 384,36 | 382,7 | |
| *Mahalalepis resima* | Middle Devonian | | Givetian | Aztec Siltstone, Antarctica | Sallan & Coates 2010; Young *et al*. 1992 | 387,7 | 382,7 | |
| *Mandageria fairfaxi* | Upper Devonian | | Famennian | Mandagery Sandstone, Canowindra, New South Wale, Australia | Johanson & Ahlberg 1997 | 367,76 | 363,33 | |
| *Marsdenichthys longioccipitus* | Upper Devonian | | Frasnian | Mt. Howitt, Victoria, Australia | Long 1985 ; Holland *et al*. 2010 | 382,7 | 379,2 | |
| *Medoevia lata* | Upper Devonian | | Famennian | Unknown locality, Russia | Lebedev 1995 | 363,33 | 358,9 | |
| *Megalichthys mullisoni* | Upper Devonian | | Famennian | Red Hill site, Duncannon Member, Catskill Formation, Clinton County, Pennsylvania, USA | Downs & Daeschler 2020 | 363,33 | 358,9 | |
| *Miguashaia bureaui* | Upper Devonian | | Frasnian | Escuminac Formation, Miguasha, Quebec, Canada | Cloutier 1996; Schultze 1973 | 379,2 | 375,7 | |
| *Miguashaia grossi* | Middle Devonian | | Givetian | Gauja and Lode Formations, Latvia | Forey *et al*. 2000 | 384,36 | 382,7 | |
| *Nasogaluakus chorni* | Lower Devonian | | Emsian | Bear Rock Formation, Anderson River, Northwest Territories, Canada | Schultze 2000 | 407,6 | 393,3 | |
| *Ngamugawi wirngarri* | Upper Devonian | | Frasnian | Gogo Formation, Australia | Clement *et al*. 2024 | 382,7 | 379,2 | |
| *Notorhizodon mackelveyi* | Middle Devonian | | Givetian | Mount Ritchie, Mount Crean, Mount Metschel, Mount Warren, Aztec Siltstone, Antarctica | Sallan & Coates 2010 | 387,7 | 382,7 | |
| *Obruchevichthys gracilis* | Upper Devonian | | Famennian | Skujaine river, Tervete Formation, Latvia | Sallan & Coates 2010; Vorobyeva 1977 | 372,2 | 367,76 | |
| *Oervigia nordica* | Upper Devonian | | Famennian | Britta Dal Formation, Celsius Bjerg Group, East Greenland | Sallan & Coates 2010; Lehman 1959 | 363,33 | 358,9 | |
| *Onychodus hopkinsi* | Middle Devonian | | Eifelian | Delaware Limestone, Ohio, USA | Newberry 1857 | 393,3 | 387,7 | |
| *Onychodus jandemarrai* | Upper Devonian | | Frasnian | Gogo Formation, Australia | Andrews *et al*. 2006 | 382,7 | 379,2 | |
| *Onychodus ortoni* | Upper Devonian | | Famennian | Ohio Shale, Huron Member, Ohio, USA; West Falls Group, New-York, USA | Newberry, 1889 | 372,2 | 358,9 | |
| *Onychodus sigmoides* | Middle Devonian | | Eifelian | Cedar Valley Limestone, Milwaukee Formation, Iowa, USA | Sallan & Coates 2010; Newberry 1857 | 393,3 | 387,7 | |
| *Orlovichthys limnatus* | Upper Devonian | | Famennian | Tugenevo beds/Dankoc-Lebedyan beds, Zadonckian/Plavsk Regional stage, central Devonian field, Oryol, Metsensk district, Russia; Ketleri outcrop, Ketleri Formation, Venta River, Latvia | Sallan & Coates 2010; Krupina 1980 | 363,33 | 358,9 | |
| *Osteolepis macrolepidotus* | Middle Devonian | | Eifelian | Sandwick Fish Bed, Cruday Quarry, Orkney Mainland | Newman 2005; Agassiz 1835 | 389,56 | 387,7 | |
| *Osteolepis panderi* | Middle Devonian | | Givetian | Spital Flagstone Formation, Upper Flagstone Group of the Middle Old Red Sandstone, Caithness | Hamilton & Trewin 1994 ; Dineley & Metcalf 1999 (p171); Pander 1860 | 387,7 | 386,03 | |
| *Palaeospondylus gunni* | Middle Devonian | | Eifelian | Sandwick fish bed in Orkney, Orcadian basin, Scotland; Achanarras Quarry, Caithness, Scotland | Sallan & Coates 2010; Newman & den Blaauwen 2010; Traquair 1890 | 389,56 | 387,7 | |
| *Panderichthys rhombolepis* | Middle Devonian | | Givetian | Lode Formation, Estonia | Boisvert *et al*. 2008 (supp.) ; Cloutier *et al*. 2020 (supp.); Gross 1930 | 384,36 | 382,7 | |
| *Panderichthys stolbowi* | Upper Devonian | | Frasnian | Schelonian beds at the river Siasj near Stolbovo, Russia | Vorobyeva 1960 | 382,7 | 372,2 | |
| *Paraglyptolepis karksiensis* | Middle Devonian | | Givetian | Burnietki Regional Stage, Karksi, Estonia | Vorobyeva 1987 | 387,7 | 382,7 | |
| *Parapanderichthys stolbovi* | Upper Devonian | | Frasnian | Stolbovo, Syas River, Dubniki beds, Sargayevian region stage, Russia | Sallan & Coates 2010; Vorobyeva 1967 | 375,7 | 372,2 | |
| *Parmastega aelidae* | Upper Devonian | | Famennian | Sosnogorsk Formation, Russia | Beznosov *et al*. 2019 | 372,2 | 367,76 | |
| *Pentlandia macroptera* | Middle Devonian | Givetian | | Last House Formation, John O’Groats Sandstone, Caithness, Scotland | Challands & den Blaauwen 2017; Traquair 1888 | 387,3 | 384,99 |  |
| *Phaneropleuron andersoni* | Upper Devonian | | Famennian | Yellow Sandstone of Dura Den | Traquair 1871; Huxley 1859 | 367,76 | 363,33 | |
| *Pillararhynchus longi* | Upper Devonian | | Frasnian | Gogo Formation, Australia | Sallan & Coates 2010; Campbell & Barwick 1990 | 382,7 | 379,2 | |
| *Pinnalongus saxoni* | Middle Devonian | | Eifelian | Brim Ness near Thurso, Caithness, Scotland | Newman & den Blaauwen 2007 | 391,43 | 389,56 | |
| *Platycephalichthys bishoffi* | Upper Devonian | | Frasnian | Stolbovo, Syas River, Dubniki beds, Sargayevian region stage, main Devonian field, northwestern Russia | Sallan & Coates 2010; Vorobyeva 1959 | 379,2 | 375,7 | |
| *Platycephalichthys skuensis* | Upper Devonian | | Famennian | Skujaine river, Tervete Formation, Latvia | Sallan & Coates 2010 | 367,76 | 363,33 | |
| *Platyethmoidea antarctica* | Middle Devonian | | Givetian | Mount Ritchie and Mount Crean, Lashly Range, Aztec Siltstone, Antarctica | Sallan & Coates 2010; Young *et al*. 1992 | 386,03 | 384,36 | |
| *Porolepis brevis* | Lower Devonian | | Pragian | Spitsbergen, Norway | Mondéjar-Fernández *et al*. 2021; Jarvik 1937 | 410,8 | 407,6 | |
| *Porolepis elongata* | Lower Devonian | | Pragian | Woodbay Formation, Spitsbergen, Norway | Jarvik 1942, 1972; Clément 2002 | 410,8 | 409,73 | |
| *Porolepis foxi* | Lower Devonian | | Pragian | Mulga Downs Group, New South Wales, Australia | Johanson *et al*. 2013 | 408,67 | 387,7 | |
| *Porolepis hefteri* | Lower Devonian | | Pragian | Siegen Formation, Rhenisch Massif, Germany | Gross 1935; Clément 2002 | 408,7 | 407,6 | |
| *Porolepis kureikensis* | Lower Devonian | | Lochkovian | Kureika and Razvedochnaya Formation, Tareya river, Siberia, Russia | Vorobyeva 1963; Clément 2022 | 419,2 | 410,8 | |
| *Porolepis posnaniensis* | Upper Devonian | | Emsian | Mimerdalen Subgroup, Spitzbergen, Norway; Placoderm sandstone, Podlazie, Holy Cross Mountains, Poland | Kade 1858; Clément 2002 | 402,83 | 358,9 | |
| *Porolepis siegenensis* | Lower Devonian | | Pragian | Siegen Formation, Rhenisch Massif, Germany | Gross 1936; Clément 2002 | 408,7 | 407,6 | |
| *Porolepis spitsbergensis* | Lower Devonian | | Pragian | Woodbay Formation, Spitsbergen, Norway | Jarvik 1942, 1972; Clément 2002 | 410,8 | 409,73 | |
| *Porolepis taimyrica* | Lower Devonian | | Lochkovian | Kureika and Razvedochnaya Formation, Tareya river, Siberia, Russia | Vorobyeva 1963; Clément 2022 | 419,2 | 410,8 | |
| *Porolepis* sp. | Lower Devonian | | Emsian | Placoderm sandstone, Podlazie, Holy Cross Mountians, Poland | Wilk 2023 | 402,83 | 393,3 | |
| *Proceratodus wagneri* | Upper Devonian | | Famennian | Cleveland Shale Member, Ohio Shale, Cleveland, Ohio, USA | Sallan & Coates 2010; Newberry 1889 | 363,33 | 358,9 | |
| *Pseudosauripterus anglicus* | Upper Devonian | Frasnian | | Brown Clee Hill, England | Ball *et al*. 1961; Woodward 1888 | 382,7 | 372,2 |  |
| *Quebecius quebecensis* | Upper Devonian | Frasnian | | Escuminac Formation, Miguasha, Quebec, Canada | Cloutier & Schultze 1996; Whiteaves 1889 | 379,2 | 375,7 |  |
| *Rhinodipterus secans* | Middle Devonian | | Givetian | Lode Formation, Gauja, Latvia | Sallan & Coates 2010; Gross 1956 | 384,36 | 382,7 | |
| *Rhinodipterus* sp. | Middle Devonian | | Eifelian | Gerolstein, Germany | Sallan & Coates 2010; Gross 1956 | 389,56 | 386,03 | |
| *Rhinodipterus stolbovi* | Upper Devonian | | Frasnian | Stolbovo, Syas River, Dubniki beds, Sargayevian region stage, Russia | Sallan & Coates 2010; Krupina 1995 | 379,2 | 375,7 | |
| *Rhinodipterus ulrichi* | Middle Devonian | | Givetian | Upper Plattenkalk, Heiligenstock quarry, Bergisch-Gladbach, Germany | Friedman 2007; Schultze 1969 | 384,36 | 379,2 | |
| *Rhynchodipterus elginensis* | Upper Devonian | | Famennian | Rosebrae Beds, Elgin, Scotland | Ahlberg *et al*. 2001 ; Friedman 2007; Säve-Söderbergh 1937 | 372,2 | 358,9 | |
| *Sauripterus anglicus* | Upper Devonian | | Famennian | Woodhill Bay Fish Bed, Upper Old Red Sandstone, Portishead, Avon, England | Sallan & Coates 2010; Woodward 1891 | 367,76 | 363,33 | |
| *Sauripteris taylori* | Upper Devonian | | Famennian | Powys Curve, Catskill Formation, Pennsylvania, USA | Hall 1843 | 363,33 | 358,9 | |
| *Scaumenacia curta* | Upper Devonian | | Frasnian | Escuminac Formation, Miguasha, Quebec, Canada | Cloutier 1996; Whiteaves 1881 | 379,2 | 375,7 | |
| *Sengoerichthys ottoman* | Upper Devonian | | Frasnian | Armutgözlek Tepe, Kemer, Turkey | Janvier *et al*. 2007 | 382,7 | 372,2 | |
| *Serenichthys kowiensis* | Upper Devonian | | Famennian | Witpoort Formation, South Africa | Gess & Coates 2015 | 363,33 | 358,9 | |
| *Shoshonia arctopteryx* | Middle Devonian | | Givetian | Beartooth Butte, Jefferson Formation, Wyoming, USA | Friedman *et al*. 2007 | 387,7 | 372,2 | |
| *Soederberghia groenlandica* | Upper Devonian | | Famennian | Aina Dal Formation, Celsius Bjerg Group, East Greenland | Sallan & Coates 2010; Lehman 1959 | 363,33 | 358,9 | |
| *Soederberghia simpsoni* | Upper Devonian | | Famennian | Mandagery Sandstone, Canowindra, New South Wales, Australia ; Aina Dal Formation East Greenland | Ahlberg *et al*. 2001 | 367,76 | 363,33 | |
| *Spodichthys buetleri* | Upper Devonian | | Frasnian | Rodebjerg Formation/Snehvide Formation, Kap Kolthoff Group, Greenland | Sallan & Coates 2010; Jarvik 1985 | 382,7 | 379,2 | |
| *Straitonia* sp. | Middle Devonian | | Givetian | Cedar Valley Limestone, Milwaukee Formation, Iowa | Sallan & Coates 2010; Thomson 1966 | 384,36 | 375,7 | |
| *Strunius walteri* | Middle Devonian | | Givetian | Upper Plattenkalk, Heiligenstock quarry, Bergisch-Gladbach near Cologne, Nordrhein-Westfalen (North Rhine-Westphalia), Germany | Upeniece 1995 ; Jessen dans Moy-Thomas 1971; Schultze & Cloutier 1996; Jessen 1966 | 384,36 | 379,2 | |
| *Styloichthys changae* | Lower Devonian | | Lochkovian | Xitun Formation, Qujing, East Yunnan, China | Zhu & Yu 2002; Friedman 2007; Zhao & Zhu 2010 | 413,6 | 410,8 | |
| Tetrapod indet. 1 | Upper Devonian | Famennian | | Greenland | Ahlberg 2024 | 363,33 | 358,9 |  |
| Tetrapod indet. 2 | Upper Devonian | | Famennian | Greenland | Ahlberg 2024 | 363,33 | 358,9 | |
| Tetrapod indet. 3 | Upper Devonian | | Famennian | Greenland | Ahlberg 2024 | 363,33 | 358,9 | |
| Tetrapod indet. 4 | Upper Devonian | | Famennian | Greenland | Ahlberg 2024 | 363,33 | 358,9 | |
| Tetrapod indet. 5 | Upper Devonian | | Famennian | Greenland | Ahlberg 2024 | 363,33 | 358,9 | |
| Trackway 1 | Middle Devonian | | Eifelian | Greenland | Ahlberg 2024 | 393,3 | 391,43 | |
| Trackway 2 | Middle Devonian | | Givetian | Valentia Island, Ireland | Stössel 1995 | 387,7 | 382,7 | |
| Trackway 3 | Upper Devonian | | Frasnian | St. Finan's Sandstone Formation at Tooreen in St. Finan's Bay, Ireland | Higgs & Meere 2024 | 379,2 | 375,7 | |
| Trackway 4 | Upper Devonian | | Frasnian | Genoa River Beds, Victoria, Australia | Warren & Wakefield 1972 | 382,7 | 372,2 | |
| *Thursius macrolepidotus* | Middle Devonian | | Eifelian | Lybster Flagstone Formation, Westerdale Quarry, Orcadian Basin, Scotland | Newman & den Blaauwen 2007; Sedgwick & Murchison 1829 | 393,3 | 389,56 | |
| *Thursius minor* | Upper Devonian | | Frasnian | Rodebjerg Formation/Snehvide Formation, Kap Kolthoff Group, Greenland | Sallan & Coates 2010; Jarvik 1985 | 382,7 | 375,7 | |
| *Thursius pholidotus* | Middle Devonian | | Eifelian | Orcadian Basin, Scotland | Traquair 1888 | 393,3 | 389,56 | |
| *Tiktaalik roseae* | Upper Devonian | | Frasnian | Okse Bay Group, middle part of the Fram Formation, Nunavut Territory, Canada | Daeschler *et al*. 2006 ; Shubin *et al*. 2013 | 382,7 | 379,2 | |
| *Tinirau clackae* | Middle Devonian | | Givetian | USA, Nevada, Simpson Park Mountains north of the Denay Valley, UCMP locality V74084 | Swartz 2012 | 384,36 | 382,7 | |
| *Tristichopterus alatus* | Middle Devonian | | Givetian | Eday Flagstone/John O' Groats Sandstone Group of South Ronaldsay, Orkney | Newman 2005 ; Egerton 1861 | 386,03 | 384,36 | |
| *Tulerpeton curtum* | Upper Devonian | | Famennian | Andreyevka-2, Khovanshshina Beds, Zovalzkh Formation, Tula, Siberia | Sallan & Coates 2010; Lebedev 1984 | 363,33 | 358,9 | |
| *Tungsenia paradoxa* | Lower Devonian | | Pragian | Posongchong Formation, Zhaotong, Yunnan Province, China | Lu *et al*. 2012 | 410,8 | 407,6 | |
| *Ventalepis ketleriensis* | Upper Devonian | | Famennian | Lnyanaya Formation, Central-European Russia; Ketleri Formation, Varkali Member, Venta River, Latvia | Schultze 1980; Lebedev & Lukševičs 2018; Lukševičs & Zupins 2004 | 366,33 | 358,9 | |
| *Ventastega curonica* | Upper Devonian | | Famennian | Ketleri outcrop, Ketleri Formation, Venta River, Latvia | Sallan & Coates 2010; Ahlberg *et al*. 1994 | 363,33 | 358,9 | |
| *Vorobjevaia dolonodon* | Middle Devonian | | Givetian | Mount Crean, Lashly Range, Antarctica | Young *et al*. 1992 | 386,03 | 384,36 | |
| *Yambira thomsoni* | Upper Devonian | | Famennian | Hunter Siltstone, Hervey Group, Grenfell, New South Wales, Australia | Sallan & Coates 2010; Johanson & Ritchie 2000 | 363,33 | 358,9 | |
| *Ymeria denticulata* | Upper Devonian | Famennian | | South side of Celsius Bjerg, Ymer Ø, North-East Greenland; Celsius Bjerg Group, Greenland | Clack *et al*. 2012 | 372,2 | 358,9 |  |
| Carboniferous | | | | | | | |  |
| *Acheroniscus caledoniae* | Mississippian | | Serpukhovian | Burghlee Ironstone, Limestone Coal Group, Loanhead, Midlothian, Scotland | Sallan & Coates 2010; Carroll 1969 | 328,33 | 325,76 | |
| *Actiobates peabodyi* | Pennsylvanian | | Kasimovian | Garnett Quarry of Kansas, USA | Schoch & Milner 2014 | 307 | 303,7 | |
| *Adamanterpeton ohioensis* | Pennsylvanian | | Moscovian | Linton, Ohio, USA | Milner & Sequeira 1998 | 310,7 | 307,5 | |
| *Adelogyrinus simorhynchus* | Mississippian | | Serpukhovian | Burghlee Ironstone, Limestone Coal Group, Loanhead, Midlothian, Scotland; Dora Bone Bed, Limestone Bone Bed, Cowdenbeath, Fife, Scotland | Sallan & Coates 2010; Watson 1929 | 328,33 | 323,2 | |
| *Allenypterus montanus* | Mississippian | | Serpukhovian | Bear Gulch Limestone, Heath Formation, Montana, USA | Lund & Lund 1985; Melton 1969 | 325,76 | 323,2 | |
| *Aemilia stellata* | Pennsylvanian | | Gzhelian | Graham Formation, Texas, USA | Mondéjar-Fern&ez *et al*. (in review) | 303,7 | 302,1 | |
| *Anthracobamus fayoli* | Pennsylvanian | | Gzhelian | Commentry, France | Werneburg 2019; Thevenin, 1906 | 303,5 | 298,9 | |
| *Askerichthys heintzi* | Pennsylvanian | | Bashkirian | Semsvik in Asker west of Oslo, Tanum Formation in the Asker Group, Norway | Borgen & Nakrem 2016 | 317,86 | 307 | |
| *Balanerpeton woodi* | Mississippian | | Viséan | East Kirkton, West Lothian, Scotland | Milner & Sequeira, 1993 | 330,9 | 330,3 | |
| *Baphetes bohemicus* | Pennsylvanian | | Moscovian | Nýřany, Czech Republic | Miller *et al*. 2009; Fritsch 1889 | 310,7 | 307,5 | |
| *Barameda decipiens* | Mississippian | | Tournaisian | Devil's Plain Formation, Mansfield group, Victoria, Australia | Long 1989 ; Cloutier *et al*. 2020 (supp.); Woodward 1906 | 358,9 | 354,83 | |
| *Barameda mitchelli* | Mississippian | | Tournaisian | Broken river and Bridge Creek, Mansfield, Victoria, Australia | Long 1989 ; Jeffery 2012; Holl& *et al*. 2007 | 358,9 | 354,83 | |
| *Caerorhachis bairdi* | Mississippian | | Serpukhovian | Burghlee Ironstone, Limestone Coal Group, Loanhead, Midlothian, Scotland | Sallan & Coates 2010; Holmes & Carroll 1977 | 328,33 | 325,76 | |
| *Caridosuctor populosum* | Mississippian | | Serpukhovian | Bear Gulch Limestone, Heath Formation, Montana, USA | Lund & Lund 1984; Torino *et al*. 2021 | 325,76 | 323,2 | |
| *Casineria kiddi* | Mississippian | | Viséan | Cheese Bay Shrimp Bed, Gullane Formation, Calciferous Sandstone Series, East Lothian, Scotland | Sallan & Coates 2010; Paton *et al*.1999 | 336,16 | 330,9 | |
| *Claradosymblema narrienense* | Mississippian | | Viséan | Raymond Formation of the Officer Basin, Queensland, Australia | Clement *et al*. 2021; Fox *et al*. 1995 | 346,7 | 336,16 | |
| *Coelacanthopsis curta* | Mississippian | | Viséan | Calciferous Sandstone Series, Scotland | Forey 1998; Traquair 1905 | 346,7 | 330,9 | |
| *Coelacanthus abdenensis* | Mississippian | | Serpukhovian | Loanhead Ironstone, Edge Coal Group, Scotland | Traquair 1903 | 330,9 | 328,33 | |
| *Coelacanthus hindi* | Mississippian | | Serpukhovian | England | Wellburn 1902 | 330,9 | 319,9 | |
| *Coelacanthus* sp. | Pennsylvanian | | Gzhelian | Ukraine | Chabakov 1927 | 303,5 | 302,5 | |
| *Coelacanthus* sp. | Pennsylvanian | | N/A | Coal Measures, Nova Scotia, Canada | Fetcher 1884 | 323,2 | 298,9 | |
| *Coelacanthus spinatus* | Mississippian | | Serpukhovian | England | Wellburn 1902 | 330,9 | 319,9 | |
| *Coelacanthus stensioei* | Mississippian | | Serpukhovian | Germany and Belgium | Aldinger 1931 | 330,9 | 328,33 | |
| *Coelacanthus tuberculatus* | Mississippian | | Serpukhovian | England | Wellburn 1902 | 330,9 | 319,9 | |
| *Coelacanthus woodwardia* | Mississippian | | Serpukhovian | England | Wellburn 1902 | 330,9 | 319,9 | |
| *Crassigyrinus scoticus* | Mississippian | | Viséan | Gilmerton Blackband Ironstone, Scotland; Dora Bone Bed, Limestone Bone Bed, Fife, Scotland | Sallan & Coates 2010; Watson 1929 | 336,16 | 328,33 | |
| *Ctenodus interruptus* | Mississippian | | Viséan | Powgree Burn, Ayrshire, Scotland; Broxburn Shale, Midlothian, Scotland; Loanhead No. 2 Ironstone and Burghlee Pit Ironstone, Midlothian, Scotland | Sallan & Coates 2010; Sharp & Clack 2013; Barkas 1869 | 341,43 | 328,33 | |
| *Delatitia breviceps* | Mississippian | | Tournaisian | Broken River fish fauna, Devil’s Plain Formation, Mansfield Group, Mansfield Basin, Victoria, Australia | Sallan & Coates 2010; Woodward 1906 | 358,9 | 354,83 | |
| *Dolichopareias disjectins* | Mississippian | | Viséan | Burdiehouse limestone, Edinburgh, Scotland | Sallan & Coates 2010; Watson 1929 | 341,43 | 336,16 | |
| *Doragnathus woodi* | Mississippian | | Viséan | Inchkeith, Upper Oil Shale Group, Firth of Forth, Fife, Scotland; Burghlee Ironstone, Limestone Coal Group, Loanhead, Midlothian, Scotland; Dora Bone Bed, Limestone Bone Bed, Cowdenbeath, Fife, Scotland | Sallan & Coates 2010; Smithson 1980 | 336,16 | 328,33 | |
| *Duffichthys mirabilis* | Upper Devonian | | Frasnian | Scat Craig Beds, Elgin, Scotland | Ahlberg 1992 | 375,7 | 372,2 | |
| *Durialepis edentatus* | Lower Devonian | | Emsian | Klerf Formation, Westphalia, Germany | Mondéjar-Fernández *et al*. 2021; Otto 2007 | 407,6 | 402,83 | |
| *Edenopteron keithcrooki* | Upper Devonian | | Famennian | Boyds Tower and Hegarty bay, New South Wales, Australia | Young *et al*. 2019; Young *et al*. 2013 | 363,33 | 358,9 | |
| *Eldeceeon rolferi* | Mississippian | | Viséan | East Kirkton Limestone, West Lothian Oil Shale Formation, Bathgate, Lothian, Scotland | Sallan & Coates 2010; Smithson 1993 | 336,16 | 330,9 | |
| *Eoherpeton watsoni* | Mississippian | | Viséan | Gilmerton Blackband Ironstone, Scotland; Burghlee Ironstone, Limestone Coal Group, Loanhead, Midlothian, Scotland; Dora Bone Bed, Limestone Bone Bed, Cowdenbeath, Fife, Scotland | Sallan & Coates 2010; Panchen 1975 | 341,43 | 328,33 | |
| *Eucritta melanolimnetes* | Mississippian | | Viséan | East Kirkton Limestone, West Lothian Oil Shale Formation, Bathgate, Lothian, Scotland | Sallan & Coates 2010; Clack 1998 | 336,16 | 330,9 | |
| *Ganopristodus lobatus* | Mississippian | | Viséan | Burdiehouse limestone, Edinburgh, Scotland | Sallan & Coates 2010; Agassiz 1844 | 341,43 | 336,16 | |
| *Ganopristodus splendens* | Mississippian | | Viséan | Gilmerton Blackband Ironstone, Gilmerton, Scotland; Burghlee Ironstone, Limestone Coal Group, Loanhead, Midlothian, Scotland | Sallan & Coates 2010; Traquair 1881 | 341,43 | 328,33 | |
| *Gephyrostegus bohemicus* | Pennsylvanian | | Moscovian | Plattenkohle of Humboldt Mine, Nýřany, Plzeň-Manětín Basin, Czech Republic | Jaekel 1902; Klembara *et al*. 2014 | 309,73 | 307 | |
| *Hadronector donbairdi* | Mississippian | | Serpukhovian | Bear Gulch Limestone, Heath Formation, Montana, USA | Lund & Lund 1984; Torino *et al*. 2021 | 325,76 | 323,2 | |
| *Latvius porosus* | Mississippian | | Tournaisian | Albert Formation, New Brunswick, Canada | Greiner 1977 ; Mickle 2017 | 358,9 | 346,7 | |
| *Lethiscus stocki* | Mississippian | | Viséan | Wardie Shale, Lower Lothian Group, Scotland | Sallan & Coates 2010; Wellstead 1982 | 336,16 | 330,9 | |
| *Letognathus hardingi* | Mississippian | | Tournaisian | Horton Bluff and Albert Formations, Bay of Fundy, Canada | Sallan & Coates 2010; Dawson 1868 | 358,9 | 350,76 | |
| *Lochmocercus aciculodontus* | Mississippian | | Serpukhovian | Bear Gulch Limestone, Heath Formation, Montana, USA | Lund & Lund 1984; Torino *et al*. 2021 | 325,76 | 323,2 | |
| *Loxomma allmanni* | Mississippian | | Viséan | Gilmerton Blackband Ironstone, Scotland | Sallan & Coates 2010; Huxley 1862 | 336,16 | 330,9 | |
| *Megalichthys hibberti* | Mississippian | | Serpukhovian | Millstone Grit, Summit, Lancashire, England | Sallan & Coates 2010; Agassiz 1835 | 325,76 | 323,2 | |
| *Megalichthys intermedius* | Pennsylvanian | | Moscovian | Coal Measures, Knowles Ironstone shale, Fenton, Staffordshire, England | Woodward 1891; Borgen & Nakrem 2016 | 315,2 | 307 | |
| *Megalichthys laticeps* | Mississippian | | Viséan | Burdiehouse limestone, Edinburgh, Scotland | Traquair 1884 | 341,43 | 336,16 | |
| *Microbrachis pelikani* | Pennsylvanian | | Moscovian | Nýřany, Czech Republic | Fritsch 1875 | 309,73 | 307 | |
| *Ophiderpeton kirktonense* | Mississippian | | Viséan | East Kirkton Limestone, West Lothian Oil Shale Formation, Scotland | Sallan & Coates 2010; Milner 1994 | 336,16 | 330,9 | |
| *Ossinodus pueri* | Mississippian | | Viséan | Middle Paddock Tetrapod Unit, Ducabrook Formation, Drummond Basin, Queensland, Australia | Sallan & Coates 2010; Warren & Turner 2004 | 341,43 | 336,16 | |
| *Papposaurus traquair* | Mississippian | | Serpukhovian | Burghlee Ironstone, Limestone Coal Group, Loanhead, Midlothian, Scotland | Sallan & Coates 2010; Watson 1914 | 328,33 | 325,76 | |
| *Pholidogaster pisciformis* | Mississippian | | Viséan | Burdiehouse limestone, Edinburgh, Scotland | Sallan & Coates 2010; Huxley 1862 | 341,43 | 336,16 | |
| *Polyosteorhynchus simplex* | Mississippian | | Serpukhovian | Bear Gulch Limestone, Heath Formation, Montana, USA | Lund & Lund 1984; Torino *et al*. 2021 | 325,76 | 323,2 | |
| *Proterogyrinus* sp. | Mississippian | | Serpukhovian | Dora Bone Bed, Limestone Bone Bed, Cowdenbeath, Fife, Scotland | Sallan & Coates 2010; Romer 1970 | 325,76 | 323,2 | |
| *Rhabdoderma alderingi* | Mississippian | | Serpukhovian | Clwyd, Wales | Forey 1998; Moy-Thomas 1937 | 330,9 | 323,2 | |
| *Rhabdoderma ardrossense* | Mississippian | | Viséan | Calciferous Sandstone Series, Fifeshire, Scotland | Forey 1998; Moy-Thomas 1937 | 346,7 | 330,9 | |
| *Rhabdoderma elegans* | Mississippian | | Serpukhovian | Linton, Ohio, USA | Forey 1998; Menning *et al*. 2001; Newberry 1856 | 330,9 | 307,26 | |
| *Rhabdoderma exiguum* | Pennsylvanian | | Moscovian | Francis Creek Shale, Mazon Creek, Illinois, USA | Clements *et al*. 2019; Eastman 1902 | 309,73 | 307 | |
| *Rhabdoderma huxleyi* | Mississippian | | Viséan | Calciferous Sandstone, Glencartholm Volcanic Group, Dumfrisshire, Scotland | Forey 1998; Traquair 1881 | 341,43 | 336,16 | |
| *Rhabdoderma newelli* | Pennsylvanian | | Kasimovian | Stanton Formation, Rock Lake Shale Member, Garnett, Kansas, USA | Echols 1963; Schultze 1995; Hibbard 1933 | 307 | 303,7 | |
| *Rhabdoderma tinglyense* | Pennsylvanian | | Bashkirian | Yorkshire, England | Forey 1998; Davis 1884 | 317,86 | 310,7 | |
| *Rhizodopsis rankini* | Pennsylvanian | | Bashkirian | Upper Drumgray Coal shale, Wester Bracco, South Lanarkshire, Scotland | Elliott 2023 | 323,2 | 315,2 | |
| *Rhizodus hibberti* | Mississippian | | Viséan | Strathclyde Group, Calciferous Sandstone Series from Crail, Fife, Scotland; Wardie shale, Scotland | Agassiz 1843 | 341,43 | 330,9 | |
| *Sagenodus copeanus* | Pennsylvanian | | Kasimovian | Montceau-les-mines, France ; Hamilton Quarry, Kansas, USA | Olive *et al*. 2012; Williston 1899 | 307 | 303,7 | |
| *Sagenodus inequalis* | Mississippian | | Serpukhovian | Millstone Grit, Summit, Lancashire, England | Sallan & Coates 2010; Owen 1867 | 325,76 | 323,2 | |
| *Sagenodus quinquecostatus* | Mississippian | | Serpukhovian | Gilmerton Blackband Ironstone, Gilmerton, Scotland; Burghlee Ironstone, Limestone Coal Group, Loanhead, Midlothian, Scotland | Sallan & Coates 2010; Traquair 1883 | 330,9 | 328,33 | |
| *Screbinodus ornatus* | Mississippian | | Viséan | Venturefair Pit near Gilmerton, Edinburgh | Jeffery 2012; Traquair 1878 | 336,16 | 330,9 | |
| *Silvanerpeton miripedes* | Mississippian | | Viséan | East Kirkton Limestone, West Lothian Oil Shale Formation, Bathgate, Lothian, Scotland | Sallan & Coates 2010; Clack 1994 | 336,16 | 330,9 | |
| *Spathicephalus mirus* | Mississippian | | Serpukhovian | Burghlee Ironstone, Limestone Coal Group, Loanhead, Midlothian, Scotland; Dora Bone Bed, Limestone Bone Bed, Cowdenbeath, Fife, Scotland | Sallan & Coates 2010; Watson 1929 | 328,33 | 323,2 | |
| *Strepsodus sauroides* | Pennsylvanian | | Moscovian | Hannah Pit, Newsham, Northumberland, England | Binney 1841; Jeffery 2004 | 312,46 | 309,73 | |
| *Tuditanus punctulatus* | Pennsylvanian | | Moscovian | Linton, Ohio, USA | Cope 1875 | 310,7 | 307,5 | |
| *Westlothiana lizziae* | Mississippian | | Viséan | East Kirkton Limestone, West Lothian Oil Shale Formation, Bathgate, Lothian, Scotland | Sallan & Coates 2010; Smithson & Rolfe 1990 | 336,16 | 330,9 | |

**Table S2.** List of the 70 basal sarcopterygian species included in disparity analyses. For each species, we indicated in which analyses it was included.

| Species | Group | Epoch | Age | Aquatic habitat | Precise palaeoenvironment | Full body | Cheek | Skull roof |
| --- | --- | --- | --- | --- | --- | --- | --- | --- |
| Devonian | | | | | | | | |
| *Acanthostega gunarri* | Tetrapoda | Upper Devonian | Famennian | Freshwater | Alluvial plain |  |  | x |
| *Barwickia downunda* | Dipnoi | Upper Devonian | Frasnian | Freshwater | Other freshwater | x |  | x |
| *Beelarongia patrichae* | Osteolepiform | Upper Devonian | Frasnian | Freshwater | Other freshwater |  | x |  |
| *Bruehnopteron murphyi* | Osteolepiform | Middle Devonian | Givetian | Marine | Other marine |  | x |  |
| *Cabonnichthys burnsi* | Osteolepiform | Upper Devonian | Famennian | Freshwater | Oxbow lake/meandering river | x | x | x |
| *Canowindra grossi* | Osteolepiform | Upper Devonian | Famennian | Freshwater | Oxbow lake/meandering river |  | x | x |
| *Diplocercides heiligenstockiensis* | Actinistia | Middle Devonian | Givetian | Marine | Lagoon |  | x |  |
| *Dipnorhynchus sussmilchi* | Dipnoi | Lower Devonian | Emsian | Marine | Reef |  |  | x |
| *Dipterus valenciennesi* | Dipnoi | Middle Devonian | Eifelian | Freshwater | Calm freshwater lake | x |  | x |
| *Durialepis edentatus* | Porolepiform | Lower Devonian | Emsian | Marine | Coastal marine |  | x | x |
| *Edenopteron keithcrooki* | Osteolepiform | Upper Devonian | Famennian | Marine | Other marine |  | x | x |
| *Elpistostege watsoni* | Tetrapoda | Upper Devonian | Frasnian | Estuary | Estuary |  |  | x |
| *Eusthenodon waengsjoei* | Osteolepiform | Upper Devonian | Famennian | Freshwater | Alluvial plain |  | x | x |
| *Eusthenopteron foordi* | Osteolepiform | Upper Devonian | Frasnian | Estuary | Estuary | x | x | x |
| *Fleurantia denticulata* | Dipnoi | Upper Devonian | Frasnian | Estuary | Estuary | x |  | x |
| *Gavinia syntrips* | Actinistia | Upper Devonian | Frasnian | Freshwater | Other freshwater |  | x |  |
| *Glyptolaemus kinnairdi* | Osteolepiform | Upper Devonian | Famennian | Freshwater | Dynamic freshwater lake | x |  |  |
| *Glyptolepis groenlandica* | Porolepiform | Middle Devonian | Eifelian | Freshwater | Alluvial plain |  | x |  |
| *Glyptolepis leptopterus* | Porolepiform | Middle Devonian | Eifelian | Freshwater | Calm freshwater lake | x |  |  |
| *Gogonasus andrewsae* | Osteolepiform | Upper Devonian | Frasnian | Marine | Reef |  |  | x |
| *Gooloogongia loomesi* | Rhizodontida | Upper Devonian | Famennian | Freshwater | Oxbow lake/meandering river | x |  | x |
| *Griphognathus sculpta* | Dipnoi | Middle Devonian | Givetian | Freshwater | Lagoon | x |  | x |
| *Griphognathus whitei* | Dipnoi | Upper Devonian | Frasnian | Marine | Reef |  |  | x |
| *Gyroptychius agassizi* | Osteolepiform | Middle Devonian | Eifelian | Freshwater | Calm freshwater lake | x | x | x |
| *Heddleichthys dalgleisiensis* | Osteolepiform | Upper Devonian | Famennian | Freshwater | Dynamic freshwater lake | x | x |  |
| *Heimenia ensis* | Porolepiform | Lower Devonian | Emsian | Freshwater | Alluvial plain | x |  |  |
| *Holoptychius jarviki* | Porolepiform | Upper Devonian | Frasnian | Estuary | Estuary | x | x | x |
| *Holoptychius nobilissimus* | Porolepiform | Upper Devonian | Famennian | Freshwater | Dynamic freshwater lake | x |  |  |
| *Howidipterus donnae* | Dipnoi | Upper Devonian | Frasnian | Freshwater | Other freshwater | x |  | x |
| *Ichthyostega stensioei* | Tetrapoda | Upper Devonian | Famennian | Freshwater | Alluvial plain |  |  | x |
| *Jarvikina wenjukowi* | Osteolepiform | Upper Devonian | Frasnian | Estuary | Estuary |  | x |  |
| *Koharalepis jarviki* | Osteolepiform | Middle Devonian | Givetian | Freshwater | Alluvial plain |  | x | x |
| *Laccognathus panderi* | Porolepiform | Middle Devonian | Givetian | Freshwater | Lagoon |  | x | x |
| *Mandageria fairfaxi* | Osteolepiform | Upper Devonian | Famennian | Freshwater | Oxbow lake/meandering river | x | x |  |
| *Marsdenichthys longioccipitus* | Osteolepiform | Upper Devonian | Frasnian | Freshwater | Other freshwater |  | x | x |
| *Miguashaia bureaui* | Actinistia | Upper Devonian | Frasnian | Estuary | Estuary | x | x | x |
| *Ngamugawi wirngarri* | Actinistia | Upper Devonian | Frasnian | Marine | Reef |  | x | x |
| *Onychodus jandemarrai* | Onychodontida | Upper Devonian | Frasnian | Marine | Reef |  | x | x |
| *Osteolepis macrolepidotus* | Osteolepiform | Middle Devonian | Eifelian | Freshwater | Calm freshwater lake | x | x | x |
| *Osteolepis panderi* | Osteolepiform | Middle Devonian | Givetian | Freshwater | Calm freshwater lake | x |  |  |
| *Panderichthys rhombolepis* | Elpistostegalia | Middle Devonian | Givetian | Freshwater | Lagoon |  | x | x |
| *Parmastega aelidae* | Tetrapoda | Upper Devonian | Famennian | Freshwater | Lagoon |  |  | x |
| *Pentlandia macroptera* | Dipnoi | Middle Devonian | Givetian | Freshwater | Calm freshwater lake | x |  | x |
| *Pinnalongus saxoni* | Dipnoi | Middle Devonian | Eifelian | Marine | Other marine | x |  |  |
| *Porolepis brevis* | Porolepiform | Lower Devonian | Pragian | Freshwater | Alluvial plain | x | x |  |
| *Quebecius quebecensis* | Porolepiform | Upper Devonian | Frasnian | Estuary | Estuary | x | x | x |
| *Rhynchodipterus elginensis* | Dipnoi | Upper Devonian | Famennian | Freshwater | Dynamic freshwater lake | x |  |  |
| *Scaumenacia curta* | Dipnoi | Upper Devonian | Frasnian | Estuary | Estuary | x |  | x |
| *Serenichthys kowiensis* | Actinistia | Upper Devonian | Famennian | Estuary | Estuary | x | x |  |
| *Soederberghia simpsoni* | Dipnoi | Upper Devonian | Famennian | Freshwater | Oxbow lake/meandering river |  |  | x |
| *Strunius walteri* | Onychodontida | Middle Devonian | Givetian | Marine | Lagoon | x | x | x |
| *Thursius macrolepidotus* | Osteolepiform | Middle Devonian | Eifelian | Freshwater | Calm freshwater lake | x | x |  |
| *Tiktaalik roseae* | Elpistostegalia | Upper Devonian | Frasnian | Freshwater | Oxbow lake/meandering river |  |  | x |
| *Tristichopterus alatus* | Osteolepiform | Middle Devonian | Givetian | Freshwater | Calm freshwater lake | x | x |  |
| Carboniferous | | | | | | | | |
| *Allenypterus montanus* | Actinistia | Mississippian | Serpukhovian | Marine | Bay | x | x | x |
| *Anthracobamus fayoli* | Tetrapoda | Pennsylvanian | Gzhelian | Freshwater | Calm freshwater lake |  |  | x |
| *Balanerpeton woodi* | Tetrapoda | Mississippian | Viséan | Freshwater | Calm freshwater lake |  |  | x |
| *Baphetes bohemicus* | Tetrapoda | Pennsylvanian | Moscovian | Freshwater | Calm freshwater lake |  |  | x |
| *Barameda decipiens* | Rhizodontida | Mississippian | Tournaisian | Freshwater | Other freshwater |  | x | x |
| *Barameda mitchelli* | Rhizodontida | Mississippian | Tournaisian | Freshwater | Other freshwater |  | x | x |
| *Caridosuctor populosum* | Actinistia | Mississippian | Serpukhovian | Marine | Bay | x | x | x |
| *Claradosymblema narrienense* | Osteolepiform | Mississippian | Viséan | Freshwater | Lagoon |  | x | x |
| *Hadronector donbairdi* | Actinistia | Mississippian | Serpukhovian | Marine | Bay | x | x | x |
| *Latvius porosus* | Osteolepiform | Mississippian | Tournaisian | Freshwater | Calm freshwater lake |  | x |  |
| *Lochmocercus aciculodontus* | Actinistia | Mississippian | Serpukhovian | Marine | Bay | x |  |  |
| *Polyosteorhynchus simplex* | Actinistia | Mississippian | Serpukhovian | Marine | Bay | x | x | x |
| *Rhabdoderma elegans* | Actinistia | Mississippian | Serpukhovian | Freshwater | Oxbow lake/meandering river | x |  | x |
| *Rhabdoderma exiguum* | Actinistia | Pennsylvanian | Moscovian | Freshwater | Delta | x |  |  |
| *Rhizodopsis rankini* | Osteolepiform | Pennsylvanian | Bashkirian | Freshwater | Other freshwater |  | x | x |
| *Screbinodus ornatus* | Rhizodontida | Mississippian | Viséan | Freshwater | Other freshwater |  | x |  |

**Table S3.** Description of landmarks and semilandmarks for the body shape, cheek region, and skull roof analyses respectively. (L) and (R) indicates the landmarks on the left or right side of the fish when paired landmarks have been digitised.

| No. | Nature of the descriptor | Description |
| --- | --- | --- |
| Body shape | | |
| 1 | Landmark | Most anterior edge of the extrascapular |
| 2 | Landmark | Insertion of the first ray of the posterior (second) dorsal fin |
| 3 | Landmark | Insertion of the last ray of the posterior (second) dorsal fin |
| 4 | Landmark | Insertion of the first ray of the dorsal lobe of the caudal fin |
| 5 | Landmark | Most posterior tip of the caudal peduncle |
| 6 | Landmark | Insertion of the first ray of the ventral lobe of the caudal fin |
| 7 | Landmark | Insertion of the first ray of the anal fin |
| 8 | Landmark | Insertion of the last ray of the anal fin |
| 9 | Landmark | Anterior insertion of the pelvic fins |
| 10 | Landmark | Dorsal insertion of the pectoral fins |
| 11 | Landmark | Most ventral part of the pectoral girdle |
| 12-31 | Semilandmarks | Dorsal body outline from landmark 1 to 2 |
| 32-67 | Semilandmarks | Posterior dorsal fin web outline from landmark 2 to 3 |
| 68-87 | Semilandmarks | Ventral body outline from landmark 11 to 9 |
| 88-97 | Semilandmarks | Ventral body outline from landmark 9 to 7 |
| 98-207 | Semilandmarks | Caudal fin web outline from landmark 4 to 6 |

**Table S3.** Description of landmarks and semilandmarks for the body shape, cheek region, and skull roof analyses respectively. (L) and (R) indicates the landmarks on the left or right side of the fish when paired landmarks have been digitised.

| No. | | Nature of the descriptor | Description |  |
| --- | --- | --- | --- | --- |
| Cheek region | | |  |  |
| 1 | Landmark | Most anterior-dorsal margin of the lacrimal (or lacrimojugal if fused) |  |  |
| 2 | Landmark | Most anterior-ventral margin of the lacrimal (or lacrimojugal if fused) |  |  |
| 3 | Landmark | Most posterior-dorsal margin of the jugal (or lacrimojugal if fused) |  |  |
| 4 | Landmark | Most posterior-ventral margin of the jugal (or lacrimojugal if fused) |  |  |
| 5 | Landmark | Most dorsal tip of the anterior margin of the post-orbital |  |  |
| 6 | Landmark | Most ventral tip of the anterior margin of the post-orbital |  |  |
| 7 | Landmark | Tip of the anterior-dorsal angle of the squamosal |  |  |
| 8 | Landmark | Tip of the anterior-ventral angle of the squamosal |  |  |
| 9 | Landmark | Tip of the posterior-ventral angle of the squamosal |  |  |
| 10 | Landmark | Tip of the anterior-dorsal angle of the operculum |  |  |
| 11 | Landmark | Posterior extremity of the operculum |  |  |
| 12 | Landmark | Tip of the anterior-ventral angle extremity of the operculum |  |  |
| 13 | Landmark | Tip of the anterior-dorsal angle of the preopercular |  |  |
| 14 | Landmark | Tip of the posterior-dorsal angle of the preopercular |  |  |
| 15 | Landmark | Tip of the anterior-ventral angle of the preopercular |  |  |
| 16 | Landmark | Tip of the posterior-ventral angle of the preopercular |  |  |
| Skull roof | | | |  |
| 1 | | Landmark | Anterior median extremity of the parietal (L) |  |
| 2 | | Landmark | Anterior median extremity of the parietal (R) |  |
| 3 | | Landmark | Posterior lateral extremity of the parietal (L) |  |
| 4 | | Landmark | Posterior lateral extremity of the parietal (R) |  |
| 5 | | Landmark | Posterior median extremity of the parietal (L) |  |
| 6 | | Landmark | Posterior median extremity of the parietal (R) |  |
| 7 | | Landmark | Anterior median extremity of the postparietal (L) |  |
| 8 | | Landmark | Anterior median extremity of the postparietal (R) |  |
| 9 | | Landmark | Posterior lateral extremity of the postparietal (L) |  |
| 10 | | Landmark | Posterior lateral extremity of the postparietal (R) |  |
| 11 | | Landmark | Posterior median extremity of the postparietal (L) |  |
| 12 | | Landmark | Posterior median extremity of the postparietal (R) |  |
| 13 | | Landmark | Most anterior extremity of the snout |  |
| 14 | | Landmark | Most anterior junction between the tabular and the postparietal (R) |  |
| 15 | | Landmark | Anterior lateral extremity of the tabular (R) |  |
| 16 | | Landmark | Most anterior junction between the tabular and the postparietal (L) |  |
| 17 | | Landmark | Anterior lateral extremity of the tabular (L) |  |
| 18 | | Landmark | Anterior margin of the orbit (R) |  |
| 19 | | Landmark | Posterior margin of the orbit (R) |  |
| 20 | | Landmark | Dorsal margin of the orbit (R) |  |
| 21 | | Landmark | Anterior margin of the orbit (L) |  |
| 22 | | Landmark | Posterior margin of the orbit (L) |  |
| 23 | | Landmark | Dorsal margin of the orbit (L) |  |

**Table S4. Convex hulls surface area.** The N/A value corresponds to classes including two or less species, making the convex hull surface area estimation inapplicable.

|  | **Class** | | | **Surface area** | | | | | | **Number of species** | | | | |
| --- | --- | --- | --- | --- | --- | --- | --- | --- | --- | --- | --- | --- | --- | --- |
|  |  | | | **Body shape** | | | **Cheek** | **Skull roof** | | **Body shape** | | | **Cheek** | **Skull roof** |
| Epoch | Lower Devonian | | | N/A | | | N/A | N/A | | 2 | | | 2 | 2 |
|  | Middle Devonian | | | 0.0384 | | | 0.1451 | 0.1217 | | 11 | | | 11 | 9 |
|  | Upper Devonian | | | 0.0690 | | | 0.1431 | 0.1786 | | 16 | | | 17 | 24 |
|  | Mississippian | | | 0.0195 | | | 0.0581 | 0.0675 | | 6 | | | 8 | 9 |
|  | Pennsylvanian | | | N/A | | | N/A | 0.0422 | | 1 | | | 1 | 3 |
| Aquatic habitat | Estuary | | | 0.0258 | | | 0.0680 | 0.1369 | | 7 | | | 6 | 7 |
|  | Freshwater | | | 0.0743 | | | 0.0403 | 0.1632 | | 22 | | | 22 | 28 |
|  | Marine | | | 0.0314 | | | 0.2776 | 0.1453 | | 7 | | | 11 | 12 |
| More precise palaeoenvironment | Alluvial plain | | | N/A | | | 0.0044 | 0.0141 | | 2 | | | 4 | 4 |
|  | Bay | | | 0.0223 | | | 0.0265 | 0.0081 | | 5 | | | 4 | 4 |
|  | Calm freshwater lake | | | 0.0179 | | | 0.0028 | 0.0534 | | 8 | | | 5 | 7 |
|  | Coastal marine | | | - | | | N/A | N/A | | 0 | | | 1 | 1 |
|  | Dynamic freshwater lake | | | 0.0017 | | | N/A | - | | 4 | | | 1 | 0 |
|  | Estuary | | | 0.0371 | | | 0.0730 | 0.1368 | | 7 | | | 6 | 7 |
|  | Fluvial delta | | | N/A | | | - | - | | 1 | | | 0 | 0 |
|  | Lagoon | | | N/A | | | 0.1465 | 0.0842 | | 2 | | | 5 | 6 |
|  | Oxbow lake/meandering river | | | 0.0135 | | | 0.0005 | 0.0633 | | 4 | | | 3 | 6 |
|  | Reef | | | - | | | N/A | 0.1001 | | 0 | | | 2 | 5 |
| Group of early sarcopterygians | Actinistia | | | 0.0334 | | | 0.1099 | 0.0304 | | 9 | | | 9 | 7 |
|  | Dipnoi | | | 0.0094 | | | - | 0.0163 | | 9 | | | 0 | 10 |
|  | “Elpistostegalia” | | | - | | | N/A | N/A | | 0 | | | 1 | 2 |
|  | Onychodontida | | | N/A | | | N/A | N/A | | 1 | | | 2 | 2 |
|  | “Osteolepiformes” | | | 0.0095 | | | 0.0262 | 0.0228 | | 10 | | | 19 | 12 |
|  | Porolepiformes | | | 0.0160 | | | 0.0128 | 0.0108 | | 6 | | | 6 | 4 |
|  | Rhizodontida | | | N/A | | | N/A | 0.0002 | | 1 | | | 2 | 3 |
|  | Tetrapoda | | | - | | | - | 0.0382 | | 0 | | | 0 | 7 |
|  | | |  |  | |  |  | | |  | |  |  |  |

**
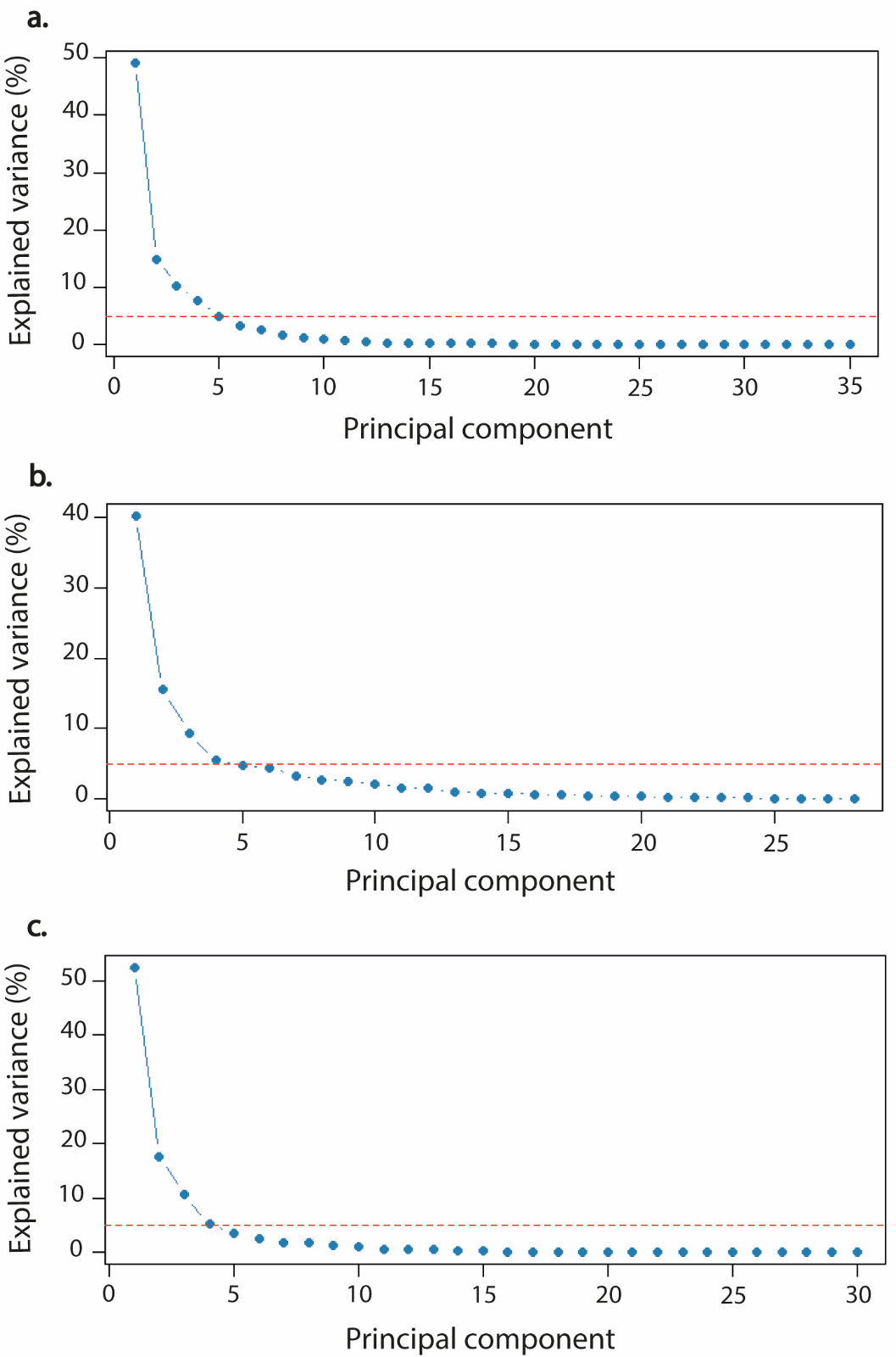
**

**Figure S1. Scree plots**. (**a**) Body shape PCA scree plot; (**b**) Cheek PCA scree plot; (**c**) Skull roof PCA scree plot. The dashed red line represents the 5% of explained variance.

**Table S5. Convex hulls weighted surface area.** Each surface area has been estimated for pairwise combination of the first five PCs and weighted by the explained variance of each pair of axes. The Simple Additive Weighting (SAW) has been used to calculate the weight of each pair of axes. The weight of the pair of axes is calculated by the average of the proportion of explained variance of each axis. The N/A value corresponds to classes including two or less species, making the convex hull surface area estimation inapplicable.

|  | **Class** | | | **Weighted surface area (SAW)** | | | | | | **Number of species** | | | | |
| --- | --- | --- | --- | --- | --- | --- | --- | --- | --- | --- | --- | --- | --- | --- |
|  |  | | | **Body shape** | | | **Cheek** | **Skull roof** | | **Body shape** | | | **Cheek** | **Skull roof** |
| Epoch | Lower Devonian | | | N/A | | | N/A | N/A | | 2 | | | 2 | 2 |
|  | Middle Devonian | | | 0.0468 | | | 0.1112 | 0.1123 | | 11 | | | 11 | 9 |
|  | Upper Devonian | | | 0.0794 | | | 0.1366 | 0.1693 | | 16 | | | 17 | 24 |
|  | Mississippian | | | 0.0199 | | | 0.0896 | 0.0722 | | 6 | | | 8 | 9 |
|  | Pennsylvanian | | | N/A | | | N/A | 0.0358 | | 1 | | | 1 | 3 |
| Aquatic habitat | Estuary | | | 0.0411 | | | 0.0636 | 0.0899 | | 7 | | | 6 | 7 |
|  | Freshwater | | | 0.0821 | | | 0.0640 | 0.1792 | | 22 | | | 22 | 28 |
|  | Marine | | | 0.0449 | | | 0.2046 | 0.1484 | | 7 | | | 11 | 12 |
| More precise palaeoenvironment | Alluvial plain | | | N/A | | | 0.0071 | 0.0165 | | 2 | | | 4 | 4 |
|  | Bay | | | 0.0175 | | | 0.0308 | 0.0115 | | 5 | | | 4 | 4 |
|  | Calm freshwater lake | | | 0.0260 | | | 0.0038 | 0.0644 | | 8 | | | 5 | 7 |
|  | Coastal marine | | | - | | | N/A | N/A | | 0 | | | 1 | 1 |
|  | Dynamic freshwater lake | | | 0.0046 | | | N/A | - | | 4 | | | 1 | 0 |
|  | Estuary | | | 0.0447 | | | 0.0837 | 0.0869 | | 7 | | | 6 | 7 |
|  | Fluvial delta | | | N/A | | | - | - | | 1 | | | 0 | 0 |
|  | Lagoon | | | N/A | | | 0.0830 | 0.1020 | | 2 | | | 5 | 6 |
|  | Oxbow lake/meandering river | | | 0.0108 | | | 0.0005 | 0.0677 | | 4 | | | 3 | 6 |
|  | Reef | | | - | | | N/A | 0.0809 | | 0 | | | 2 | 5 |
| Group of early sarcopterygians | Actinistia | | | 0.0448 | | | 0.1123 | 0.0380 | | 9 | | | 9 | 7 |
|  | Dipnoi | | | 0.0247 | | | - | 0.0199 | | 9 | | | 0 | 10 |
|  | “Elpistostegalia” | | | - | | | N/A | N/A | | 0 | | | 1 | 2 |
|  | Onychodontida | | | N/A | | | N/A | N/A | | 1 | | | 2 | 2 |
|  | “Osteolepiformes” | | | 0.0169 | | | 0.0349 | 0.0231 | | 10 | | | 19 | 12 |
|  | Porolepiformes | | | 0.0152 | | | 0.0168 | 0.0149 | | 6 | | | 6 | 4 |
|  | Rhizodontida | | | N/A | | | N/A | 0.0003 | | 1 | | | 2 | 3 |
|  | Tetrapoda | | | - | | | - | 0.0430 | | 0 | | | 0 | 7 |
|  | | |  |  | |  |  | | |  | |  |  |  |

**Table S6. Convex hulls weighted surface area.** Each surface area has been estimated for pairwise combination of the first five PCs and weighted by the explained variance of each pair of axes. The Weighted Sum of Pairwise Areas (WSPA) has been used to calculate the weight of each pair of axes. The weight of each pair of axes is calculated by multiplying the proportion of variance explained by each. The N/A value corresponds to classes including two or less species, making the convex hull surface area estimation inapplicable.

|  | **Class** | | | **Weighted surface area (WSPA)** | | | | | | **Number of species** | | | | |
| --- | --- | --- | --- | --- | --- | --- | --- | --- | --- | --- | --- | --- | --- | --- |
|  |  | | | **Body shape** | | | **Cheek** | **Skull roof** | | **Body shape** | | | **Cheek** | **Skull roof** |
| Epoch | Lower Devonian | | | N/A | | | N/A | N/A | | 2 | | | 2 | 2 |
|  | Middle Devonian | | | 0.0070 | | | 0.0166 | 0.0199 | | 11 | | | 11 | 9 |
|  | Upper Devonian | | | 0.0122 | | | 0.0189 | 0.0288 | | 16 | | | 17 | 24 |
|  | Mississippian | | | 0.0030 | | | 0.0120 | 0.0128 | | 6 | | | 8 | 9 |
|  | Pennsylvanian | | | N/A | | | N/A | 0.0061 | | 1 | | | 1 | 3 |
| Aquatic habitat | Estuary | | | 0.0062 | | | 0.0095 | 0.0171 | | 7 | | | 6 | 7 |
|  | Freshwater | | | 0.0124 | | | 0.0076 | 0.0286 | | 22 | | | 22 | 28 |
|  | Marine | | | 0.0066 | | | 0.0314 | 0.0265 | | 7 | | | 11 | 12 |
| More precise palaeoenvironment | Alluvial plain | | | N/A | | | 0.0009 | 0.0028 | | 2 | | | 4 | 4 |
|  | Bay | | | 0.0029 | | | 0.0044 | 0.0019 | | 5 | | | 4 | 4 |
|  | Calm freshwater lake | | | 0.0036 | | | 0.0005 | 0.0100 | | 8 | | | 5 | 7 |
|  | Coastal marine | | | - | | | N/A | N/A | | 0 | | | 1 | 1 |
|  | Dynamic freshwater lake | | | 0.0005 | | | N/A | - | | 4 | | | 1 | 0 |
|  | Estuary | | | 0.0070 | | | 0.0113 | 0.0171 | | 7 | | | 6 | 7 |
|  | Fluvial delta | | | N/A | | | - | - | | 1 | | | 0 | 0 |
|  | Lagoon | | | N/A | | | 0.0134 | 0.0175 | | 2 | | | 5 | 6 |
|  | Oxbow lake/meandering river | | | 0.0017 | | | 0.0001 | 0.0111 | | 4 | | | 3 | 6 |
|  | Reef | | | - | | | N/A | 0.0151 | | 0 | | | 2 | 5 |
| Group of early sarcopterygians | Actinistia | | | 0.0066 | | | 0.0157 | 0.0063 | | 9 | | | 9 | 7 |
|  | Dipnoi | | | 0.0031 | | | - | 0.0029 | | 9 | | | 0 | 10 |
|  | “Elpistostegalia” | | | - | | | N/A | N/A | | 0 | | | 1 | 2 |
|  | Onychodontida | | | N/A | | | N/A | N/A | | 1 | | | 2 | 2 |
|  | “Osteolepiformes” | | | 0.0023 | | | 0.0044 | 0.0039 | | 10 | | | 19 | 12 |
|  | Porolepiformes | | | 0.0023 | | | 0.0020 | 0.0024 | | 6 | | | 6 | 4 |
|  | Rhizodontida | | | N/A | | | N/A | 0.0000 | | 1 | | | 2 | 3 |
|  | Tetrapoda | | | - | | | - | 0.0073 | | 0 | | | 0 | 7 |
|  | | |  |  | |  |  | | |  | |  |  |  |

**Table S7. Morphological disparity analyses.** The N/A values correspond to the class including only one species, making the variance estimation inapplicable.

|  | **Class** | | | **Procrustes variance** | | | | | | **Proportion of total disparity (%)** | | | | |
| --- | --- | --- | --- | --- | --- | --- | --- | --- | --- | --- | --- | --- | --- | --- |
|  |  | | | **Body shape** | | | **Cheek** | **Skull roof** | | **Body shape** | | | **Cheek** | **Skull roof** |
| Epoch | Lower Devonian | | | 0.0122 | | | 0.0082 | 0.0566 | | 4.574 | | | 2.661 | 5.091 |
|  | Middle Devonian | | | 0.0251 | | | 0.0714 | 0.0633 | | 18.658 | | | 25.081 | 19.762 |
|  | Upper Devonian | | | 0.0350 | | | 0.0702 | 0.0607 | | 37.998 | | | 37.107 | 48.628 |
|  | Mississippian | | | 0.0318 | | | 0.1003 | 0.0456 | | 35.529 | | | 32.222 | 16.920 |
|  | Pennsylvanian | | | N/A | | | N/A | 0.0846 | | 3.241 | | | 2.929 | 9.599 |
| Aquatic habitat | Estuary | | | 0.0305 | | | 0.0828 | 0.0656 | | 14.670 | | | 15.681 | 15.603 |
|  | Freshwater | | | 0.0320 | | | 0.0397 | 0.0614 | | 49.045 | | | 31.593 | 57.557 |
|  | Marine | | | 0.0444 | | | 0.1297 | 0.0639 | | 36.285 | | | 52.726 | 26.840 |
| More precise palaeoenvironment | Alluvial plain | | | 0.0118 | | | 0.0328 | 0.0303 | | 5.184 | | | 7.063 | 5.861 |
|  | Bay | | | 0.0365 | | | 0.0797 | 0.0303 | | 33.539 | | | 30.340 | 7.774 |
|  | Calm freshwater lake | | | 0.0198 | | | 0.0154 | 0.0523 | | 13.879 | | | 5.651 | 19.761 |
|  | Coastal marine | | | - | | | N/A | N/A | | - | | | 1.675 | 2.107 |
|  | Dynamic freshwater lake | | | 0.0180 | | | N/A | - | | 8.415 | | | 1.141 | - |
|  | Estuary | | | 0.0350 | | | 0.0830 | 0.0656 | | 16.212 | | | 18.406 | 18.222 |
|  | Fluvial delta | | | N/A | | | - | - | | 3.355 | | | - | - |
|  | Lagoon | | | 0.0274 | | | 0.1129 | 0.0769 | | 5.170 | | | 21.131 | 18.067 |
|  | Oxbow lake/meandering river | | | 0.0381 | | | 0.0069 | 0.0474 | | 14.246 | | | 4.925 | 12.270 |
|  | Reef | | | - | | | 0.0771 | 0.0800 | | - | | | 9.668 | 15.938 |
| Group of early sarcopterygians | Actinistia | | | 0.0323 | | | 0.0961 | 0.0327 | | 43.890 | | | 47.927 | 12.329 |
|  | Dipnoi | | | 0.0267 | | | - | 0.0193 | | 22.945 | | | - | 28.850 |
|  | “Elpistostegalia” | | | - | | | N/A | 0.0107 | | - | | | 3.248 | 3.567 |
|  | Onychodontida | | | N/A | | | 0.0241 | 0.0333 | | 2.518 | | | 11.349 | 8.501 |
|  | “Osteolepiformes” | | | 0.0131 | | | 0.0278 | 0.0180 | | 16.966 | | | 24.257 | 14.436 |
|  | Porolepiformes | | | 0.0135 | | | 0.0347 | 0.0247 | | 9.710 | | | 10.374 | 8.662 |
|  | Rhizodontida | | | N/A | | | 0.0210 | 0.0087 | | 3.971 | | | 2.845 | 5.282 |
|  | Tetrapoda | | | - | | | - | 0.0365 | | - | | | - | 18.373 |
|  | | |  |  | |  |  | | |  | |  |  |  |


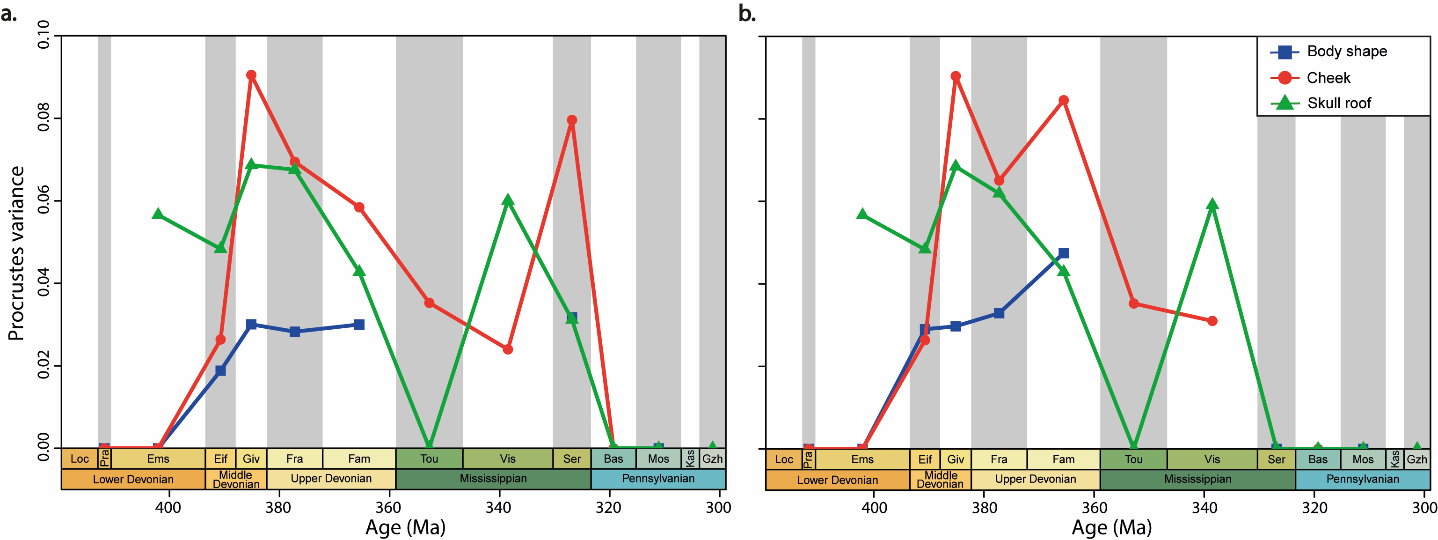


**Figure S2. Lagerstätte effect**. (**a**) Procrustes variances for each age for the complete dataset; (**b**) Procrustes variances for each age without Bear Gulch species.
